# Protocol of identical exercise programs with and without specific breathing techniques for the treatment of chronic non-specific low back pain: randomized feasibility trial with two-month follow-up

**DOI:** 10.1186/s12891-023-06434-6

**Published:** 2023-05-05

**Authors:** Jani Mikkonen, Hannu Luomajoki, Olavi Airaksinen, Liesbet Goubert, Ville Leinonen

**Affiliations:** 1 Private Practice, Mikonkatu 11, 00100 Helsinki, Finland; 2 Department of Surgery (Incl. Physiatry), Institute of Clinical Medicine, University of Eastern Finland, 70211 Kuopio, Finland; 3 ZHAW School of Health Professions, Zurich University of Applied Sciences, CH-8401 Winterthur, Switzerland; 4 Department of Experimental-Clinical and Health Psychology, Ghent University, 9000 Ghent, Belgium; 5 Department of Neurosurgery, Institute of Clinical Medicine, University of Eastern Finland, 70211 Kuopio, Finland

**Keywords:** Breathing exercise, Breathing techique, Movement control exercise, Chronic low back pain, Yoga, Pilates

## Abstract

**Background:**

Chronic low back pain (CLBP) is a leading cause of disability globally. Exercise therapies are one of the commonly prescribed treatment options for CLBP. The specific exercise therapies for CLBP most commonly target movement dysfunction, but seldom brain-based pain modulation. Exercise therapies with specific breathing techniques (SBTs) have been shown to influence and enhance brain-based structural and functional pain modulation.

**Aims and objectives:**

To assess the feasibility of the SBTs protocol, eligibility criteria, randomization, and dropout rates. To quantify the changes in patient outcome measures and choose the most relevant measure for larger-scale study. To quantify self-adherence levels to home exercise and monitor and record possible pain medication and other treatment modality usage, and adverse events during exercise.

**Design:**

A parallel randomised analyst-blinded feasibility trial with two-month follow-up.

**Outcome measures:**

Feasibility related to aims and objectives. Multiple pain- and health-related patient-reported outcome measures of pain intensity, disability, central sensitization, anxiety, kinesiophobia, catastrophising, self-efficacy, sleep quality, quality of life, and health and well-being status. Exercise adherence, pain medication and other treatment modality usage, and possible adverse events related to exercises will be monitored and recorded.

**Methods:**

Thirty participants will be randomized to movement control exercise with SBTs (15 subjects in experimental group) or movement control exercise without SBTs (15 subjects in control group) in private chiropractic practice setting with two-month follow-up. Trial registration number; NCT05268822.

**Discussion:**

The clinical difference in effectiveness between practically identical exercise programs in uniform study settings with or without SBTs has not been studied before. This study aims to inform feasibility and help determine whether progression to a full-scale trial is worthwhile.

**Supplementary Information:**

The online version contains supplementary material available at 10.1186/s12891-023-06434-6.

## Introduction

Chronic low back pain (CLBP) is a leading cause of disability globally, with enormous personal, social and economic burdens [1]. The transition to CLBP is usually preceded by several episodes of low back pain of varying lengths and intensity [2]. Biopsychosocial factors, including genetic predisposition, lifestyle factors, pain modulation factors, and several chronic disease comorbidities, are known contributory factors to the development of CLBP over time [1, 3]. Exercise therapies are one of the most commonly prescribed treatment options for CLBP, although the effect sizes are small to moderate [4]. Generally, management of CLBP is very challenging and require new, simple, more effective, less costly, and safe exercise approaches to promote more effective management strategies [5].

The most specific exercise therapies for CLBP concentrate on biophysical output mechanisms such as treatment of motor control because chronic musculoskeletal pain is associated with impaired motor control [6–9]. Among the most studied and most evidence-based and CLBP guideline–recommended approaches to assess and treat motor control are the low back movement control tests and exercises of Luomajoki et al. [10, 11]. The reliability of assessment and well-documented exercises for the treatment of movement control impairment make this exercise approach easily applicable for study with practically identically tested and treated exercise groups [10, 12–14]. The main clinical challenge from an effective pain treatment perspective is that the exercises target impaired motor function, but not well-documented abnormalities on central nociceptive structures and functional changes [15–20].

Yoga and Pilates emphasise the importance of different specific breathing techniques (SBTs) with exercise in the treatment of chronic musculoskeletal pain [21–23]. These body–mind interventions are increasingly popular, researched and implemented in healthcare settings to treat pain [23–25]. Exercises with SBTs have been shown to potentially enhance multiple brain-based structural and/or functional changes confirmed on imaging studies [26–29]. These brain-based positive changes overlap negative changes related to chronic pain seen in imaging [30–32]. In clinical studies, the effectiveness of exercises with SBTs are multifactorial, addressing multiple biopsychosocial factors contributing to the development and persistence of CLBP [33–40]. Moreover, exercises with SBTs appear safe or safer when compared to other exercise types [41, 42]. However, the mechanisms underlying the multifactorial clinical effectiveness of exercises incorporating SBTs are sparsely studied and barely understood [24, 25, 43–45]. Furthermore, studies of yoga are generally conducted with very heterogeneous SBTs protocols and include many other types of practices [46]. Because of the heterogeneity of study protocols, it is hard to determine the effect of SBTs alone on clinical outcome differences compared to exercise without SBTs for different chronic pain syndromes.

There are no previous studies that have compared the outcome of identical exercises with or without SBTs in a uniform clinical study setting to inform differences in multifactorial patient-reported outcome measures (PROMs). Hence, this study aims to inform feasibility and help determine whether progression to a full-scale trial is worthwhile. With positive outcomes from this feasibility study and following a full-scale randomized controlled trial with several therapy providers and guideline-recommended pain patient education, SBTs could become a valuable add-on to almost any specific exercise therapy treating chronic pain. SBTs can be implemented for exercise therapy in everyday clinical practice with minimal extra training for therapists, without extra costs, without any extra equipment and without extra side effects or risks of injury for participants.

### Objectives

1. To assess the feasibility of a clinical study protocol, including an SBTs protocol, eligibility criteria, randomization and dropout rates.
2. To quantify the changes in PROMs to help determine whether progression to a full-scale trial is worthwhile and to inform the choice of the most relevant and responsive PROM for a larger-scale study.
3. To quantify self-adherence levels to home exercise and to monitor possible pain medication and other treatment modality usage, as well as possible adverse events and injuries during exercise using a home diary.

## Methods

### Trial design

A parallel randomised analyst-blinded feasibility trial with two-month follow-up. The study will be carried out in a single private chiropractic clinic. The study is a single blinded as administer will not be blinded for study intervention.

Thirty participants after meeting eligibility criteria will be allocated to movement control exercises with SBTs (experimental group) or movement control exercises without SBTs (control group) with an allocation ratio of 1:1.

**Fig. 1:**
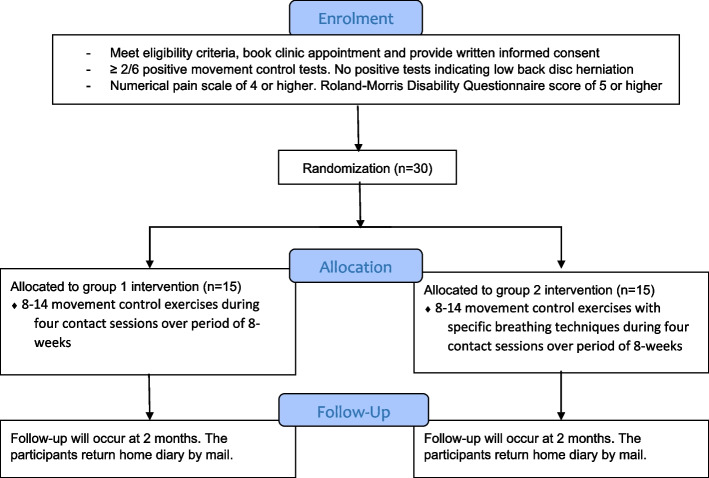
CONSORT 2010 flow diagram

### Participants

#### Eligibility criteria

The participants will be eligible for the study if they meet all inclusion and no exclusion criteria.

Inclusion Criteria:

- Males and females aged 18–68 years
- Low back pain lasting more than 3 months (pain sensation more than 3 days per week)
- A numerical pain scale of 4 or higher on a scale of 0 to 10 to prevent floor effects in outcome measurement [47]
- Roland-Morris Disability Questionnaire score of 5 or higher on a scale of 0 to 24 to prevent floor effects in outcome measurement [48]
- Physically able to perform movement control tests and provide written informed consent
-  ≥ 2/6 positive low back movement control tests as described by Luomajoki et al., which are well-documented and valid tests of movement control dysfunction on participants with CLBP [12, 13, 49]. The tests are:
  1. Waiters bow test for assessment of flexion movement control impairment
  2. Dorsal tilt of pelvis for assessment of extension movement control impairment
  3. One leg stance for assessment of lateral flexion and/or rotational movement control impairment
  4. Sitting knee extension for assessment of flexion or rotational movement control impairment
  5. Rocking backwards for assessment of flexion movement control impairment and rocking forwards for assessment of extension movement control impairment
  6. Prone lying active knee flexion assessment of extension and/or rotational movement control impairment

Exclusion Criteria:

- Any history of malignancy
- Neurological disease affecting the central nervous system (MS, dementia)
- Rheumatic disease (fibromyalgia, ankylosing spondylitis/rheumatoid arthritis)
- Chronic obstructive pulmonary disease or other diseases that affect the lungs and cause breathing problems
- Spinal surgery in the last 12 months
- A cardiac pacemaker
- Pregnancy during the data collection
- Signs and symptoms of lumbar nerve root pathology during the eligibility assessment following neurological examination.
  a Toe and heel walk three metres
  b Lower extremity reflexes
  c Sitting Slump tests
  d Femoral nerve stretches tests on side lying
  e Supine active and passive straight leg raises on ipsi- and contralateral legs

The main aim of the inclusion and exclusion criteria is to attempt to minimize the effect of intervention differences by including only participants with movement control impairment. Exclusion is based on standardized and reliable low back movement control tests by Luomajoki et al. [12, 13, 49]. Furthermore, treating specific movement control impairment with standardized exercises is an attempt to minimize difference in effect size, exercise variation and physical performance heterogeneity between the groups. A second aim of the tight eligibility criteria is to exclude known cardiovascular, respiratory, rheumatic and neurological diseases potentially affecting breathing mechanics. The eligibility criteria for the neurological examination aim to minimise group difference heterogeneity to include only participants with non-specific CLBP and exclude participants with nerve root pathology due to intervertebral disc herniation or lumbar spinal stenosis, using an established, recommended, reliable and comprehensive neurological examination [50]. Signs and symptoms of possible nerve root pathology are based on the first author’s clinical decision and participants with a high probability of nerve root involvement will be excluded.

#### Recruitment

The research advertisement for the clinical trial will be presented on the first author’s private clinic webpage. In addition, different national Finnish musculoskeletal pain and spine-related organisations, chronic pain peer support groups, and healthcare colleagues will promote the study on their web pages and social media.

Potentially eligible patients meeting the criteria for this study will be invited to read the participant information sheet and consider enrolling in the study. Enrolled participants will book eligibility assessment appointments at the first author’s clinic, where written consent for the study will be given. After written consent, neurological examination of nerve root pathology and low back movement control clinical tests as described by Luomajoki et al. [12, 13] will be carried out as described in detail above.

After the eligibility assessment, the study participants will complete study questionnaires on the webpages of Navisec Health at home. Navisec Health is a Finnish company providing an electronic platform with strong electronic authentication for data collection and storage of study questionnaires for participants. In Finland, strong electronic identification enables participants to verify their identity safely in various electronic services before filling in PROMs.

The participants meeting inclusion and exclusion criteria will be invited by email to book the first research appointment from the internet time booking calendar. We anticipate needing 120 participants with CLBP to complete the clinical eligibility assessment and the study PROMs to recruit 30 participants. The sample size of 30 participants is a general recommendation for feasibility and pilot trials [51]. The sample size calculation for this feasibility study will not be calculated, because it is not recommended when there is no previous data from previous studies of similar study setting to inform this process [52].

#### Randomization procedure

Thirty participants will be allocated by simple randomization prior to start of participant recruitment, with an allocation rate of 1:1. The random allocation list will be generated by the first author using the SPSS program. The participants will be included in the study in order of meeting the inclusion criteria and no exclusion criteria. They will subsequently book their first research appointment via the internet calendar booking system according to their timetables. Hence, the first author will be unable to decide in advance on the allocation group of the participant. Participants are blinded for intervention differences between groups.

### Outcome variables

#### Feasibility questions

1. Are eligibility criteria too inclusive or restrictive to recruit potential participants?
2. How many participants do we need to assess to recruit eligible 30 participants?
3. Are study groups balanced in numbers and demographics after simple randomization?
4. How well subjects can incorporate the SBTs protocol with movement control exercises during clinic visits?
5. How useful diary is in collection of detail of adherence for exercises, medication, usage of other therapies, and possible adverse events during exercise?
6. What is the dropout rate in this study setting?

The main feasibility criteria progress information for potential full-scale trial are introduced on Table 1.

**Table 1:** The main feasibility progression criteria

Feasibility criteria	Interpretation	Progress information to potential full-scale trial
Specific breathing protocol	Add-on breathing technique	Acceptable participants adherence of breathing protocol during clinic visits
Outcome measures	Diary data	Acceptable participants adherence of home exercises
	Patient-reported outcome measures	Clinically relevant outcomes measure differences between groups
	Patient-reported outcome measures	Clinically relevant outcome measures responsiveness in order to choose primary and secondary outcome measures for larger scale study
Eligibility criteria	Inclusive or restrictive recruitment criteria	Successful recruitment in acceptable time frame
Randomization	Simple randomization	Balance study groups in numbers and demographics

#### Outcome measures

Outcome measures are assessed on the baseline and at the two-month follow-up only in aim to avoid wasting resources if there are no relevant group differences in outcome measures. Multiple CLBP-related PROMs are included to help determine responsiveness of outcome measures and whether progression to a full-scale trial is worthwhile.

1. Numerical pain rating scale (NRPS). The NRPS is a widely used subjective assessment of pain. It is an 11-point numerical pain scale ranging from 0 (no pain) to 10 (worst imaginable) [53]. More than 1.5-point change represent minimal detectable change (MDC) [54] and a 2-point change on the NPRS represents minimal clinically important difference (MCID) in participants with low back pain [47].
2. The Roland-Morris Disability Questionnaire is a 24-item questionnaire used to evaluate CLBP-related disability. The scale ranges from 0 (no disability) to 24 (maximum low back pain-related disability) [55, 56]. The MCID difference is estimated to be a change of 2 to 3 points compared to the baseline score for low back pain patients [57].
3. The Central Sensitization Inventory (CSI) was developed as a tool for screening central sensitisation (CS) [58]. It is a two-part questionnaire in which part A contains 25 questions on CS-related symptomology using a Likert scale from 0 = never to 4 = always. The total score ranges from 0 to 100. Part B includes ‘No/Yes’ and ‘year diagnosed’ questions about previous diagnoses related to CS-related disorders. Part B of the CSI is to provide information and is not scored [59]. MDC varies from 5.9 to 8.9 between different low back pain populations [60]. The CSI has been translated into Finnish and validated among a Finnish CLBP population [61].
4. The Generalized Anxiety Disorder Assessment (GAD-7). The GAD-7 is a self-reported measure of generalised anxiety disorder–related symptoms. The items are rated over the preceding two weeks from not at all = 0 to 3 = nearly every day. Thus, the total scale ranges from 0 (the most minimal anxiety) to 21 (the most severe anxiety) [62]. The MCID score for GAD-7 is 4 [63]. The GAD-7 has been adapted and validated in Finnish [64].
5. The Tampa Scale of Kinesiophobia (TSK). The TSK is used for assessment of subjective kinesiophobia (fear of movement). It has 17 statements related to kinesiophobia, with answers ranging from ‘strongly disagree’ to ‘strongly agree’, yielding a total range from 17 (minimal kinesiophobia) to 68 (maximal kinesiophobia) [65]. The MDC score is 8 [66] and MCID score TSK is 5.5 [67] The TSK has been translated into and validated in Finnish [68].
6. The Pain Catastrophizing Scale (PCS). The PCS is used to assess the tendency to magnify the threat value of a pain stimulus. Thirteen items are scored on a Likert scale from 0 to 5, producing total scores from 0 (no catastrophising thoughts) to 52 (maximum catastrophising thoughts) [69]. The MDC score is 8 [69]. The PCS has been translated into Finnish but has not been cross-culturally validated. This study is part of its cross-cultural validation in Finnish.
7. The Pain Self-Efficacy Questionnaire (PSEQ) includes 10 items. It is developed to assess the self-efficacy that people in pain have in their daily activities. The scale ranges from 0 points (not at all confident) to 6 points (completely confident). The PSEQ is applicable to all chronic pain conditions, but has mostly been validated on CLBP populations with the MDC score is 11.5 and MCID score from 5.5 to 8.5 [70]. The PSEQ has been translated into and validated in the Finnish language [71].
8. The Pain and Sleep Questionnaire Three-Item Index (PSQ-3). This is a three-question questionnaire studying the effects of pain on sleep. The scale ranges from 0 (pain does not affect sleep) to 30 (pain has maximum effect on sleep) [72]. The PSQ-3 has been translated into Finnish and validated among a Finnish CLBP population [73].
9. The first part of the EuroQol (EQ-5D-5L) is used to assess five dimensions of health-related quality of life [74]—mobility, self-care, usual activities, pain/discomfort and anxiety/depression—on a Likert scale (0 = no problems, 1 = slight problems, 2 = moderate problems, 3 = severe problems, 4 = unable/extreme problems). The EQ visual analogue scale (EQ VAS) is the second part of the EQ-5D-5L [74]. As a standard value set has not yet been studied for the Finnish population, a value set from a Danish population was used to calculate the index value. This is recommended by the EuroQol EQ-5D-5L User Guide [75].
10. The Well-Being in Pain Questionnaire is a self-developed 11-question questionnaire to screen for the effects of pain on a person's biopsychosocial well-being using a Likert scale from 0 = never to 4 = always. Total scores range from 0 (no subjective well-being in pain) to 44 (maximum subjective well-being in pain). The questionnaire is a novel measurement developed by the first author and collaborators. This study is part of its validation.

A home diary will be used to monitor the regularity and estimate the amount of time spent in minutes on home exercises every day for the eight-week study period. Moreover, the home diary will be used to collect the participants’ use of pain medication (frequency of use, type, dose), other treatments or co-interventions for the treatment of pain (e.g. massage, chiropractic, manual therapy, physiotherapy) and possible adverse events and injuries related to the movement control exercises at home. The daily home diary will be identical for both groups.

The movement control tests will be assessed again during the last fourth clinical appointment to compare test results to those prior to the study period.

#### Shared in-clinic treatment protocol for both groups

A single clinician (the first author) will administer the assessment and treatment of movement control exercises to both groups. The first author is an experienced and long-term clinician in treating chronic musculoskeletal pain with exercises and breathing exercises. The advantage of this single-clinician study design is its ability to minimise the intervention differences and effects of contextual factors, such as clinic environment, location, architecture, interior design and therapists’ behaviour, communication and attitudes, which are well established as having a major effect on therapeutic placebo and/or nocebo effects in clinical encounters [76, 77].

After allocation to groups, each subject will be asked to attend four one-to-one contact sessions (30 min per session) on an individual basis over eight weeks. The most commonly anticipated in-clinic research appointment frequency is one week after the first session (week 2/8 in the study), two weeks later (week 4/8 in the study) and two weeks after that (week 6/8 in the study).

For both groups in the research appointments, visual demonstration, verbal guidance and/or hands-on assistance will emphasise the importance of motor learning focused on precision of movement control according to individual subject impairments. The primary goal of movement control intervention is to retrain optimal movement control and coordination of the spine, pelvis, hips and limbs to avoid ongoing nociceptive input secondary to suboptimal tissue loading [8].

The movement control exercises are intended to treat flexion (nine different exercise candidates), extension (nine different exercise candidates) and/or lateral flexion–rotational movement impairments (five different exercise candidates), as shown in Table 2. Two out of 23 exercises (camel and cat exercises) are more stretching type of exercises, in which the movement control exercise aim is to learn to return to a neutral spine after stretching. The exercises are mainly adapted from previous studies and the movements control handbook for treating CLBP by Luomajoki [14, 78, 79].

**Table 2:**
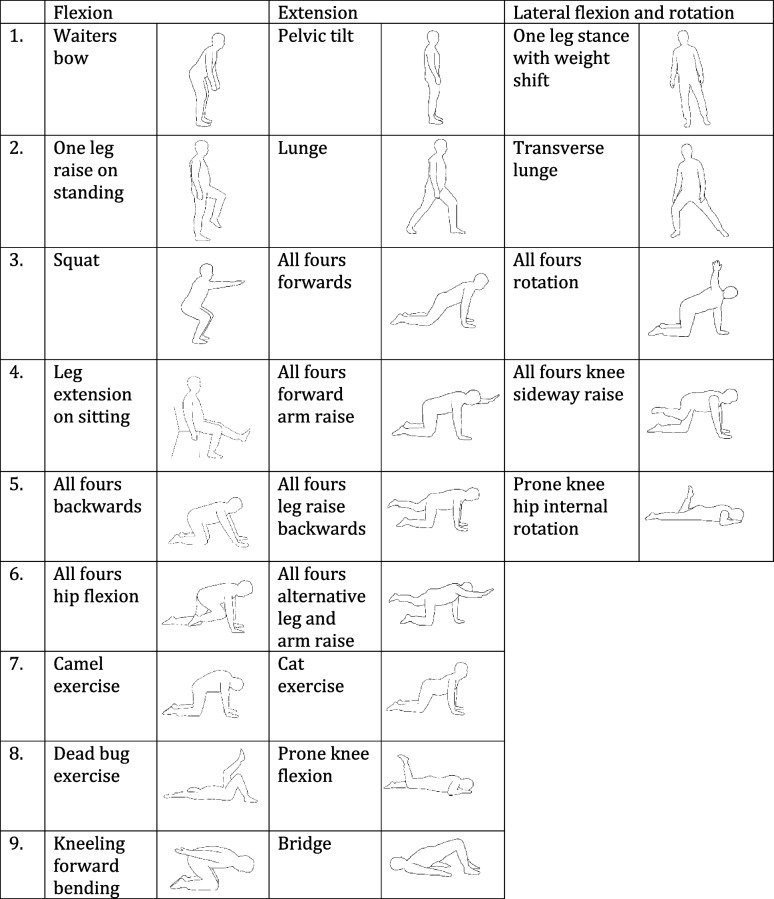
Movement control exercises

The exact exercises will be chosen according to the individual movement impairment, but similar exercises will be emphasized in both groups. Alternative exercises will mainly be used if an exercise is not applicable because of aggravation of pain or because it is physically too easy or challenging to perform. Hence, movement control exercise variation will be minimized by employing similar exercises in both study groups.

Progress in the precision of movement control will be assessed at each research appointment. There will be between three and six exercises provided in the first research appointment to treat major movement control impairment identified by low back movement control tests. Further exercises will be given according to individual needs with the expectation that there will be between 8 and 14 different exercises by the final fourth research appointment. In a situation in which there is no specific movement control impairment on any test, all exercises in the study can be applied according to the exercise protocol to ensure variation in exercises. The employed exercises, sets and repetitions will be documented in written home exercise sheets documented by the first author and a group comparison will be carried out between these in the final report.

The treatment protocol is minimalist. It is not intended to include pain neuroscience education, other therapeutic modalities or lifestyle advice. The aim is to avoid mixing treatments in order to evaluate more precisely the outcome differences of the study groups.

#### In-clinic SBTs add-on protocol for the experimental group (group 2.)

The SBTs used in the movement control exercises will follow the principles of the main therapeutic yoga styles [80–82] and breathing exercises that are the most commonly used in a healthcare setting [83]. The main practical aim of a specific breathing protocol is to keep it as simple as possible so that it can be more easily adapted by other therapists to other exercise treatment protocols in the future.

1. Breathing through the nose if it feels easy and natural
2. Mental focus on using abdominal breathing
3. Synchronization of the breathing cycle (inhale-small pause-exhale-small pause) with movement during the movement control exercises. Detailed instructions for synchronization of the breathing cycle with the movement for each exercise are provided in detail in Appendices 1, 2 and 3.

In exercises, the synchronization of the breathing cycle with movements will mainly be based on different yoga styles that incorporate the Vinyasa method, which means synchronising breathing with movement, as in this study protocol [81]. Synchronisation of the breathing cycle with movement follows the simple general spine movement rule, whereby spine movement while *exhaling* involves bending forward and relaxing the spine and while *inhaling* involves expanse, straightening the spine and/or bending backwards [81, 82]. In several movement control exercises in the protocol, there are only upper and/or lower extremity movements, with static spine movement (e.g. weight shifts on one leg standing to the other) and for these, breathing synchronization varies according to the individual movement. Also, in different yoga styles, the exact instructions for synchronisation of breathing with movement vary for different movements. This study’s version of detailed synchronisation of breathing with movement for each exercise is described in detail in Appendices 1, 2 and 3.

#### Home exercise protocol

The treatment protocol of the study emphasises home exercise because in a previous study of the mechanism of yoga on the treatment of CLBP, the biggest mediator on decreased disability was increased self-efficacy [84]. The participants in both groups will be instructed to practice the prescribed exercises as many days as possible in a week. The maximum recommended daily time for exercise will be 20 min per day. Participants will be instructed to continue any other exercises they performed before the start of the study.

The general home instructions provided on the sheets are as follows:

- Maintain a neutral lumbar spine during the exercises.
- Synchronise your breathing with the movements (Note: The breathing instructions are only included on the exercise sheets for group 2).
- Have breaks between sets and exercises according to your individual needs.
- Try to practice your exercises regularly/once a day.
- Remember to regularly update your home diary.

The home exercise sheets are accompanied by individually prescribed repetitions and sets of each exercise from the first author. The participants will have been given the possibility of contacting the first author via email or phone for further clarification of any exercise-related matter. All exercises used in the study with original written home instructions translated from Finnish with breathing instructions are included ins Appendices 1, 2 and 3. The breathing instructions comprise the only difference between the home exercise sheets; they are only provided to group 2.

The protocol of the study attempts to promote identical clinical encounters and treatments for both groups with only the difference of the SBTs add-on for group 2. Different aspects of these efforts are summarised in Table 3.

**Table 3:** Summary of efforts to promote practically identical clinical practice exercise programmes

Clinical entity	Aim
Inclusion and exclusion criteria	Include only subjects who are adults with low back pain lasting more than three months (pain sensation more than three days per week) to promote a uniform pain population
	Exclude known cardiovascular, respiratory, rheumatic and neurological diseases potentially affecting breathing mechanics and hence potentially affecting the effects of SBTs
Clinical assessment before inclusion	Include only participants with movement control impairment with exactly similar test batteries to promote homogeneity of treatment and physical performance heterogeneity between the groups
	Include only participants with non-specific CLBP, excluding participants with symptomatic nerve root pathology due to intervertebral disc herniation or lumbar spinal stenosis
Single-clinician study design	The aim is to minimize the intervention differences and effects of contextual factors of clinic settings, such as environment, interior design and therapists’ behaviour, communication and approach to treatment
Exercise	The research appointment follows a similar frequency over eight weeks with the aim of uniform treatment between groups
	There will be a similar number of exercises delivered to the subject in each clinical visit to promote uniform treatment between groups
	Clinical instructions for both groups concentrate on the treatment of movement control impairment
	The exact exercises are chosen according to individual movement impairment, but similar exercises are emphasized for all participants
	Identical general instructions for both groups
Other interventions	The aim is to exclude other evidence-based interventions in order to evaluate more precisely the outcome differences of the study groups

#### Data analysis

Descriptive statistical methods will be used. Statistical analysis will be performed using SPSS version 25 (IBM SPSS Statistics for Windows, Version 25.0. Armonk, NY: IBM Corp.). The normality of the data will be checked by Shapiro-Wilks tests. Demographics data will be shown as percentages or means with standard deviations or medians, with ranges depending on the distribution of the variables. The results of the PROMs will be presented as means (Standard deviation or 95% confidence interval, lower and upper bounds) as recommended on CONSORT 2010 statement: extension to randomised pilot and feasibility trials [85] Furthermore, we will follow the general recommendation of feasibility study sample size of 30 subjects [51].

The external data analyst, who is not involved in planning or conducting the study, will independently collect data from electronic data storage and carry out the data analysis of the comparison between groups. Moreover, the data analyst is blinded to the aims of the study and group intervention differences to increase the objectivity of the group comparison.

## Discussion

SBTs are a simple, free and safe addition to exercises to potentially give them an improved multidimensional clinical outcome. A clear and easily clinically implemented SBTs protocol is presented in this study. To date, possible clinical outcome differences with an SBTs add-on to exercises have not been studied for otherwise identical clinical settings and exercises.

The positive feasibility outcomes of this study could raise questions about the generalizability of the SBTs add-on to other exercise interventions to improve clinical outcomes. Due to the multifactorial nature of CLBP, SBTs add-on could be one more functional piece in a biopsychosocial and individualised treatment puzzle where exercise is one of the basic pieces.

The next step after a possibly favourable outcome from this feasibility study would be an evaluation of the clinical outcome of the breathing add-on in a larger randomized controlled trial study that would include the most relevant and responsive PROM(s) as primary and secondary outcome measures, clinicians with different backgrounds and experience levels, multicentre clinical settings and other guideline-recommended adjunct therapies, such as pain neuroscience education.

### Strengths and limitations

The major strength of this study is the pragmatic and easy-to-implement SBTs protocol with well-documented and practically identical exercises to inform possible outcomes related to exercise with and without SBTs. The other main strengths of the study are the thorough and clinically relevant inclusion and exclusion criteria, with blinding of data analysts in relation to group differences and the study aims, in order to increase the reliability of results. As well as strength and limitation is the single-clinician study design, which is aimed at minimizing the intervention and contextual factor differences but which also affects the objectivity of exercise delivery by a single author and the generalisability of the study results for other therapists with less experience with SBTs. A second major limitation is the lack of intervention blinding for the therapist and participants, which is not generally possible in similar exercise intervention study designs.

#### Ethical approval and trial registration

Ethical approval for the study was obtained from the Research Ethics Committee of the Northern Savo Hospital District with identification number 2131/2022 on 31st January 2022. Written informed consent will be obtained from all participants before the study. The study is conducted according to the Declaration of Helsinki. The study has been registered with ClinicalTrials.gov Identifier: NCT05268822 on 8th February 2022. CONSORT 2010 statement: Extension to randomised pilot and feasibility trials were adopted upon reporting this feasibility trial protocol (Fig. 1) [85]. The SPIRIT checklist has been implemented to improve the content and quality of this protocol [86] and the CERT template was used to improve exercise reporting [87].

## Supplementary Information

**Additional file 1.**

**Additional file 2.** 

**Additional file 3. **

## Data Availability

Not applicable (NA).
