# Supplementary material for: Protocol of identical exercise programs with and without specific breathing techniques for the treatment of chronic non-specific low back pain: randomized feasibility trial with two-month follow-up
Source: BMC Musculoskelet Disord. 2023 May 5;24:354. doi: 10.1186/s12891-023-06434-6 (PMC10161472; doi:10.1186/s12891-023-06434-6)
Supplement: Supplementary file 1 — Additional file 1. [file 12891_2023_6434_MOESM1_ESM.docx]

**Extension movement control exercises**

- **Maintain a neutral lumbar spine during the exercises.**
- **Synchronize your breathing with the movements (Note: The breathing instructions were only included on the exercise sheets for group 2, otherwise the exercise sheets were identical for both groups.)**
- **Have breaks between sets and exercises according to your individual needs.**
- **Try to practice your exercises regularly/once a day.**
- **Remember to regularly update your home diary.**

**Standing exercises**


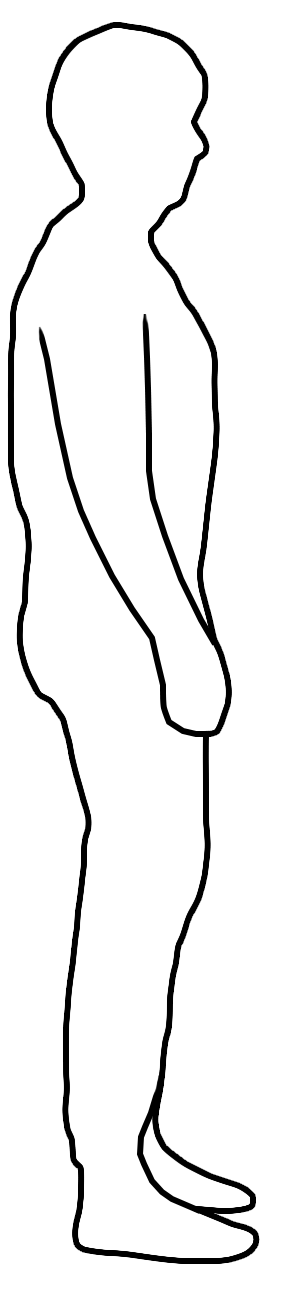


1. **Pelvic tilt.** Stand with your feet the same distance apart as your hips. Bring your tailbone forward and under as your lower spine straightens at the same time. Aim to move your tailbone, pelvis and lower back without moving your upper or lower extremities. Inhale as you tuck your tailbone under and exhale as you come back to the starting position.

Repeat movement ______ repetitions ______ sets.


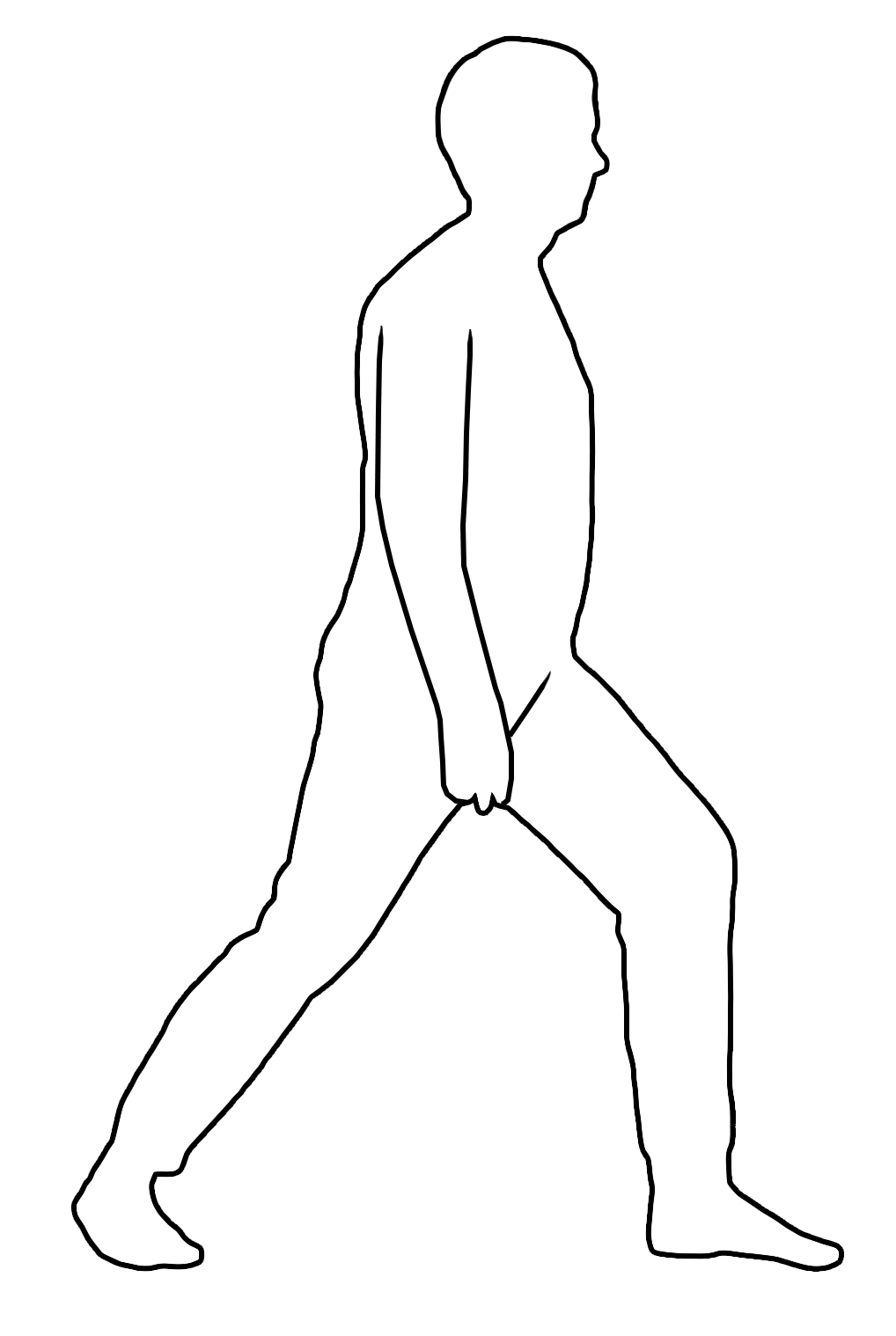


1. **Lunge.** Stand with your feet the same distance apart as your hips. Step ahead with one leg and let your front knee bend. Let your toes on your back leg point outwards. Maintain a neutral spine as you step ahead. Step back to the starting position and repeat with the other leg. Inhale as you step ahead and exhale as you come back to the starting position.

Repeat movement ______ repetitions ______ sets with each leg.

**All fours exercises**
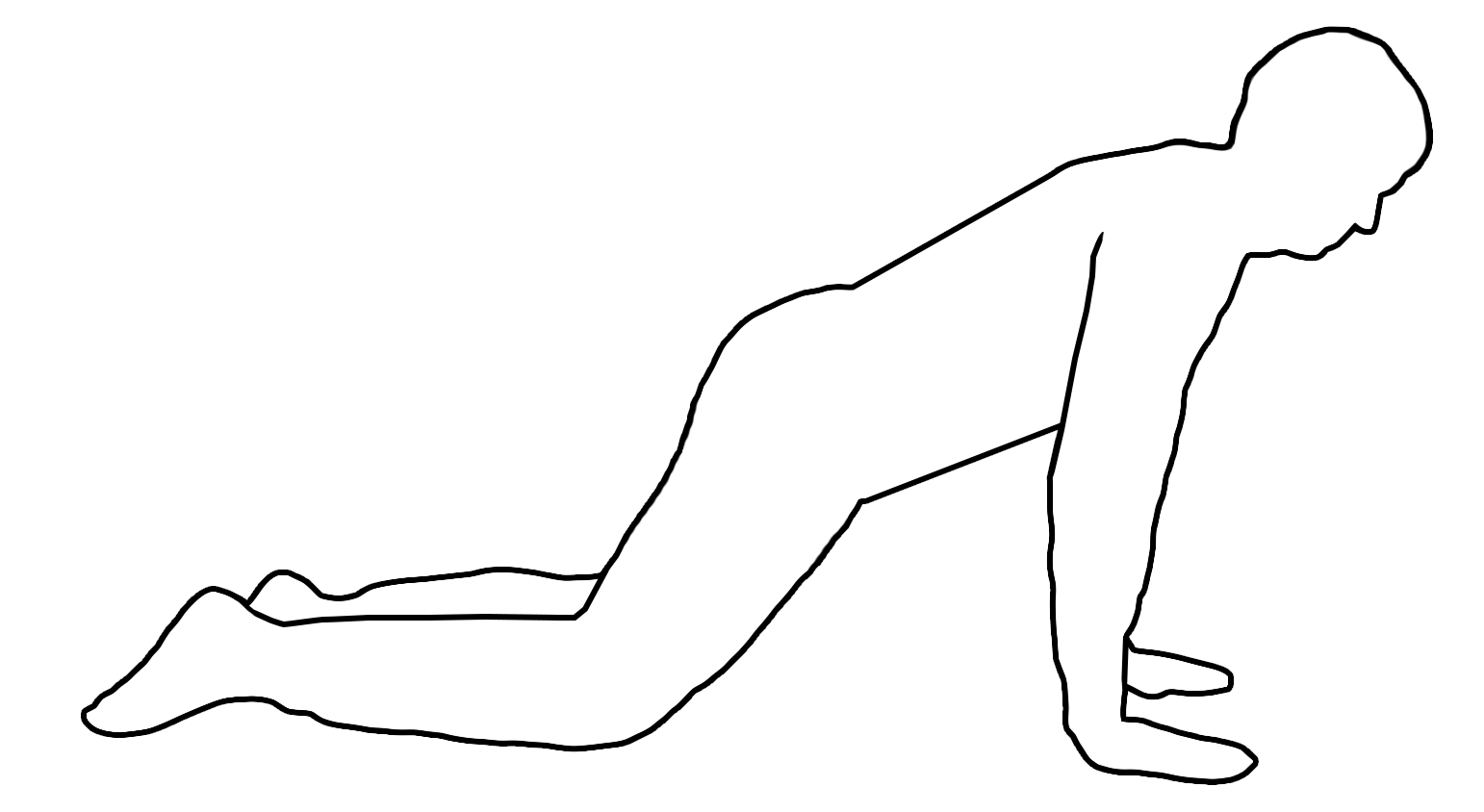


1. **All fours forwards.** Kneel on all fours with a neutral lower spine, with your hands and knees separated by the same width as your hips. Start to bring your pelvis forwards while maintaining a neutral lower spine. Inhale as you bring your pelvis forward and exhale as you come back to the starting position.

Repeat movement ______ repetitions ______ sets.

1.
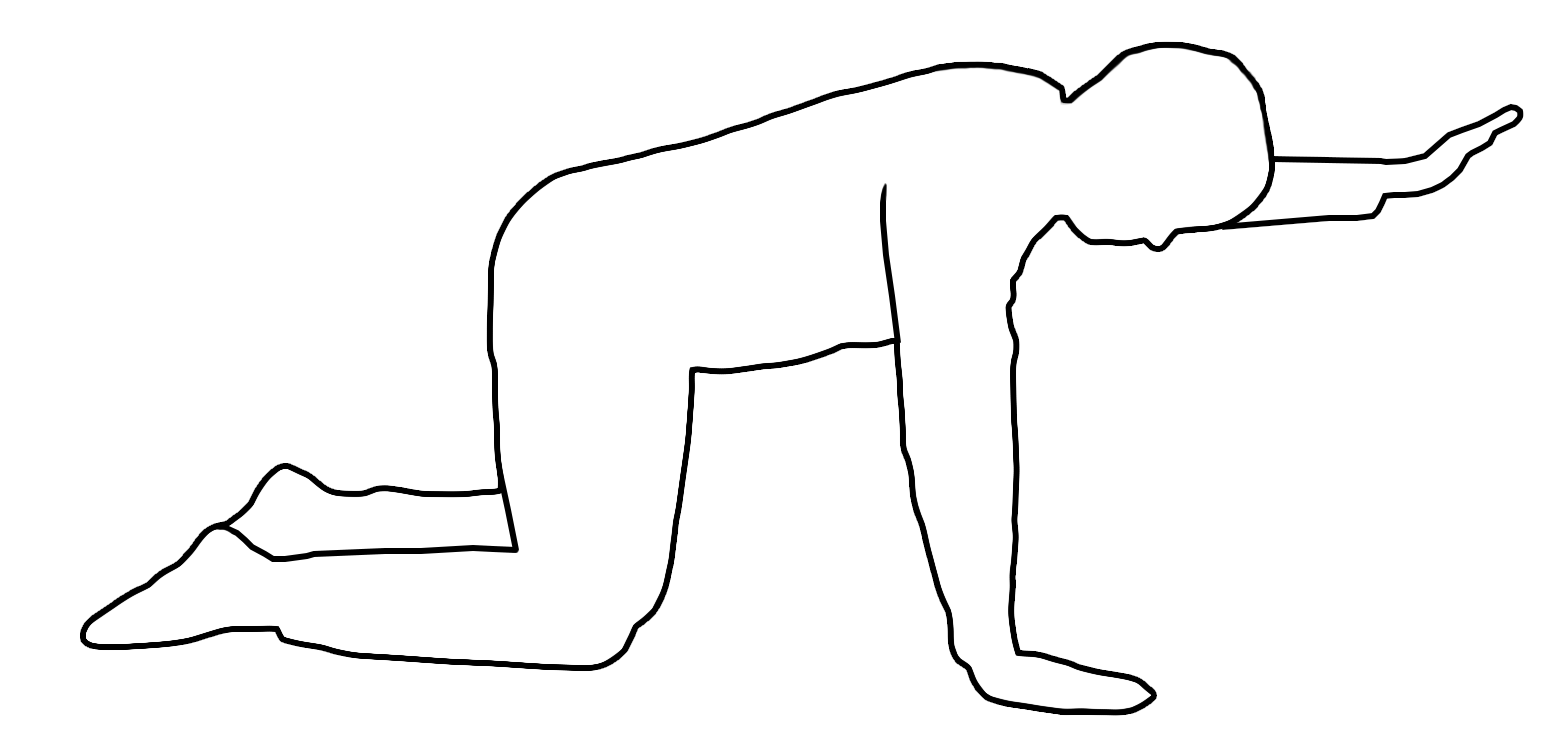
**All fours forward arm raise.** Kneel on all fours with a neutral lower spine, with your hands and knees separated by the same width as your hips. Raise one arm up, while keeping your arm straight and pointing your fingers straight forward. Inhale as you raise your arm and exhale as you come back to the starting position.

Repeat movement ______ repetitions ______ sets, alternating your arm between repetitions.

1.
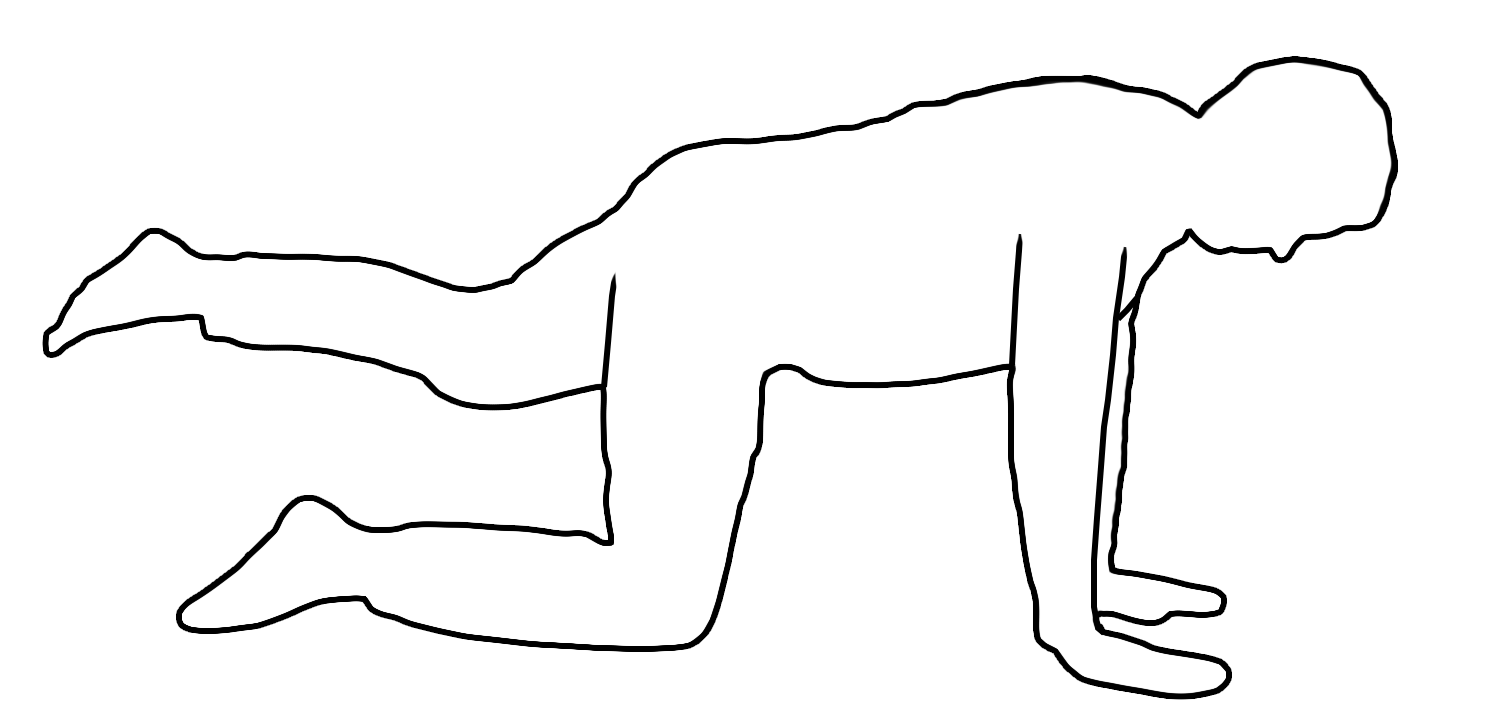
**All fours leg raise backwards.** Kneel on all fours with a neutral lower spine, with your hands and knees separated by the same width as your hips. Raise one leg up as far you can comfortably. Inhale as you raise your leg and exhale as you come back to the starting position.

Repeat movement ______ repetitions ______ sets, alternating your leg between repetitions.

1.
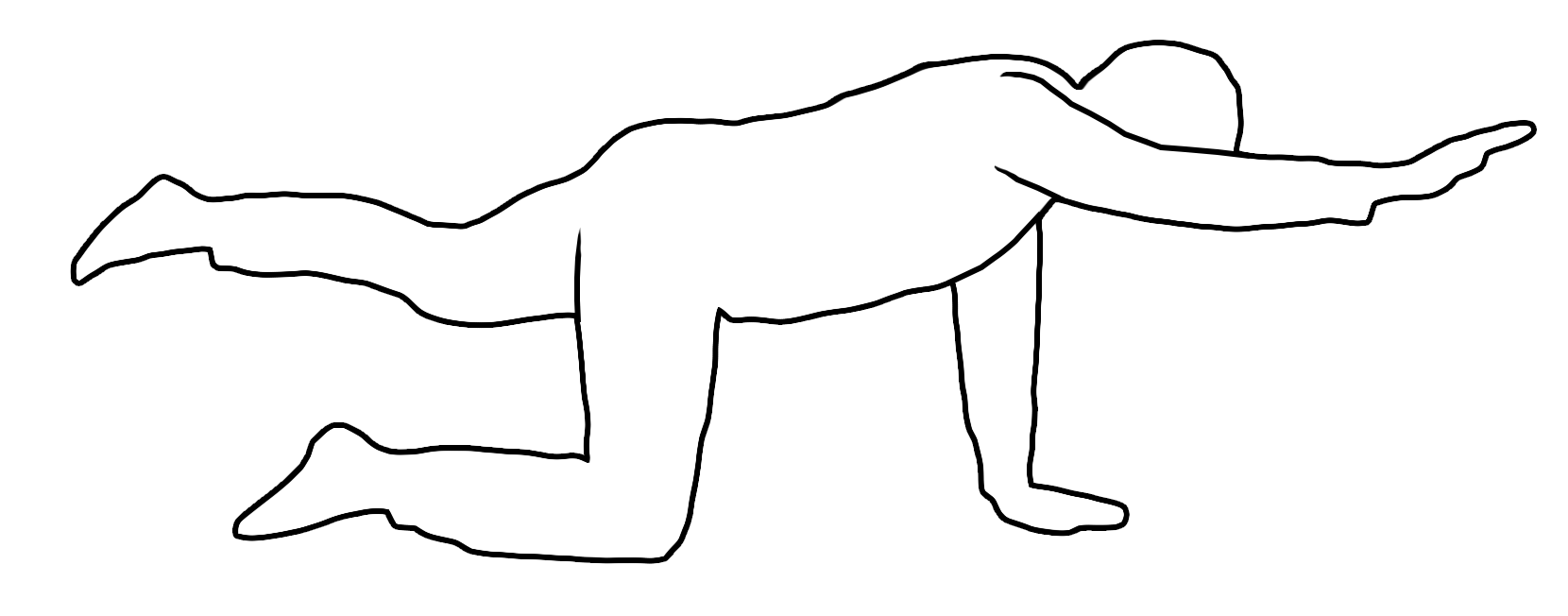
**All fours alternative leg and arm raise.** Kneel on all fours with a neutral lower spine, with your hands and knees separated by the same width as your hips. Raise one arm and the opposite leg up as far you can comfortably. Inhale as you raise your arm and leg and exhale as you come back to the starting position.

Repeat movement ______ repetitions ______ sets, alternating your arm and leg between repetitions.

1.
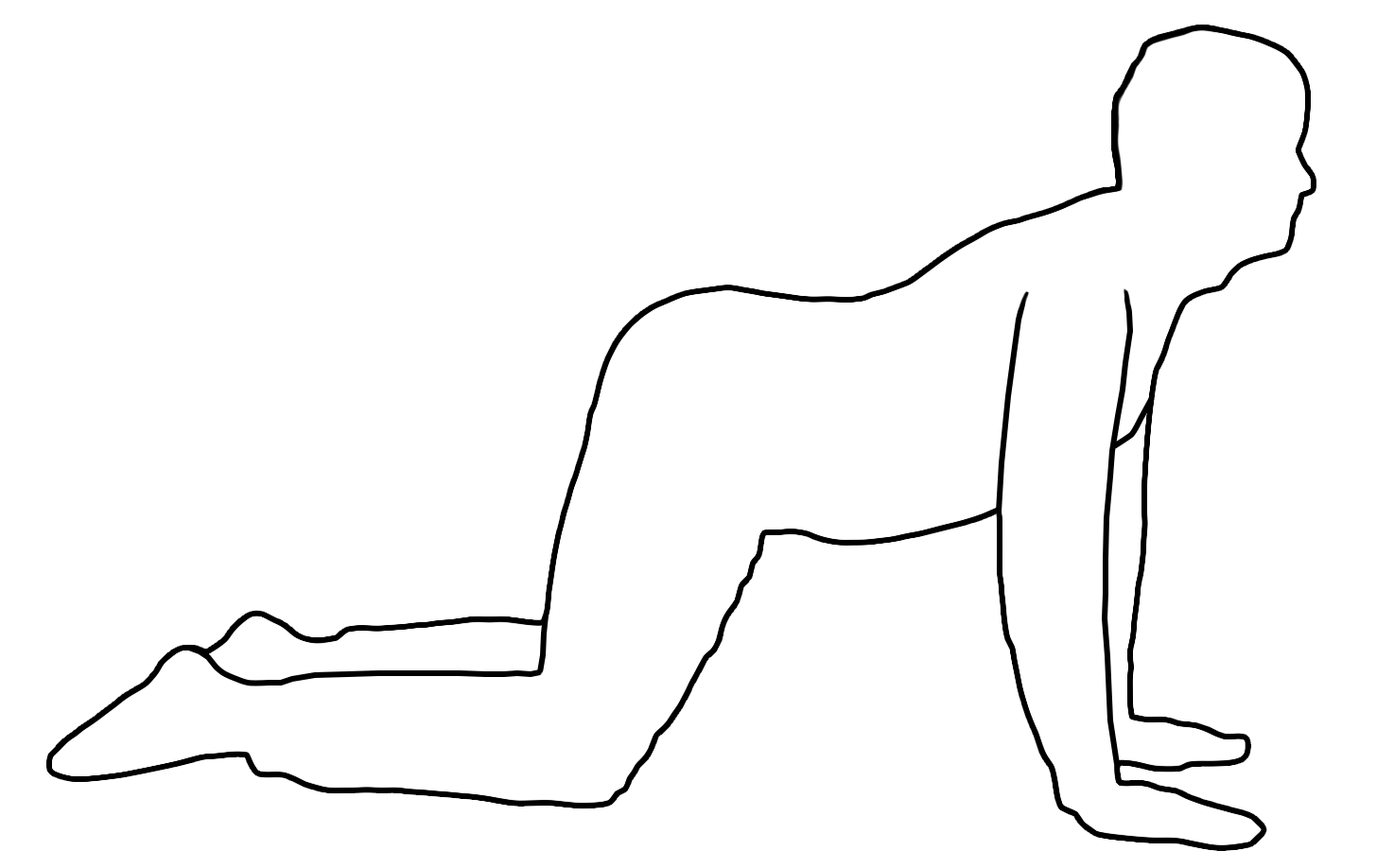
**Cat exercise.** Kneel on all fours with a neutral lower spine, with your hands and knees separated by the same width as your hips. Lift your head, relax your shoulders away from your ears, open your chest and emphasise the inward curve of your lower spine while tucking your tailbone outwards. Exhale as you open your chest and emphasise the inward curve of the lower spine and inhale as you come back to the starting position.

Repeat movement ______ repetitions ______ sets.

**Prone exercise**


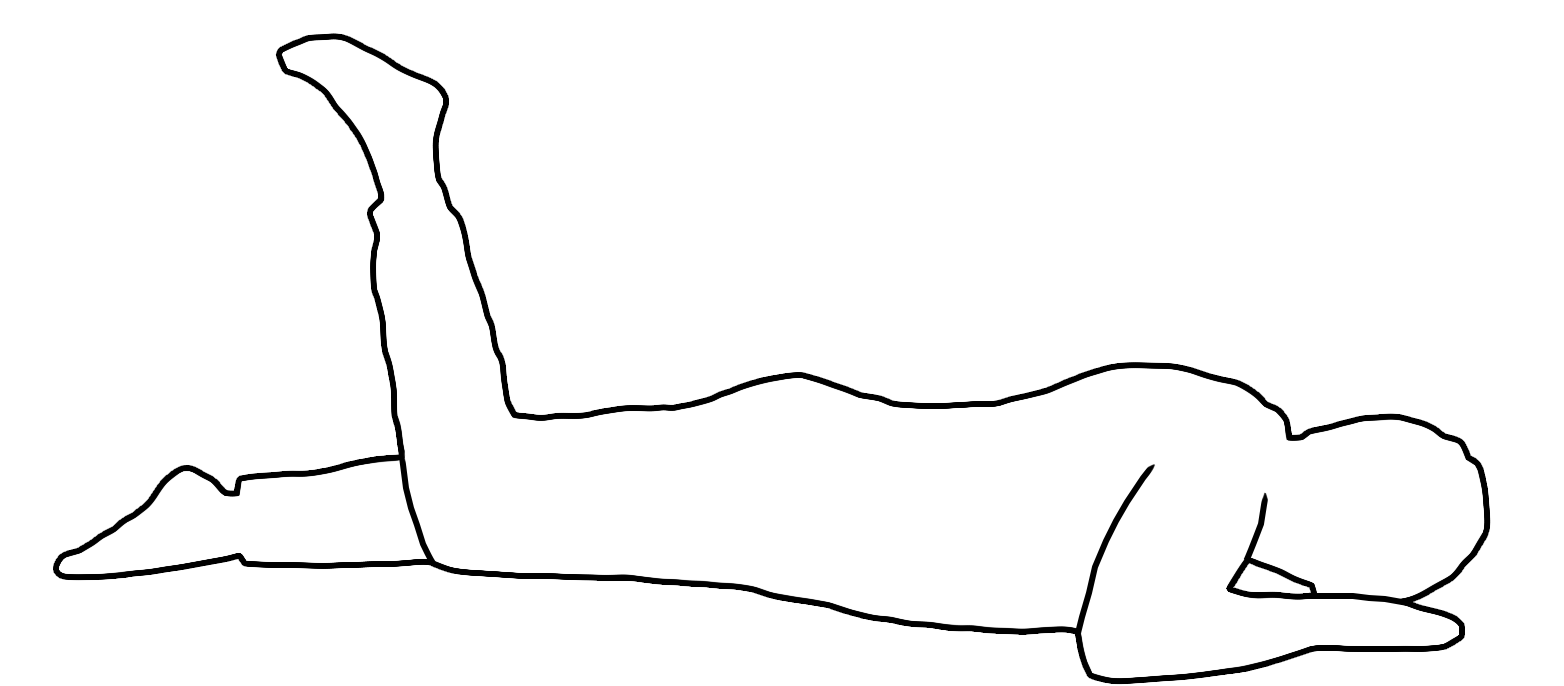
**8. Prone knee flexion.** Lie on your stomach as you maintain a neutral lower spine. Inhale as you bend your knee and exhale as you straighten your leg. Alternate legs between repetitions.

Repeat movement ______ repetitions ______ sets, alternating your leg between repetitions.

**Supine exercises**


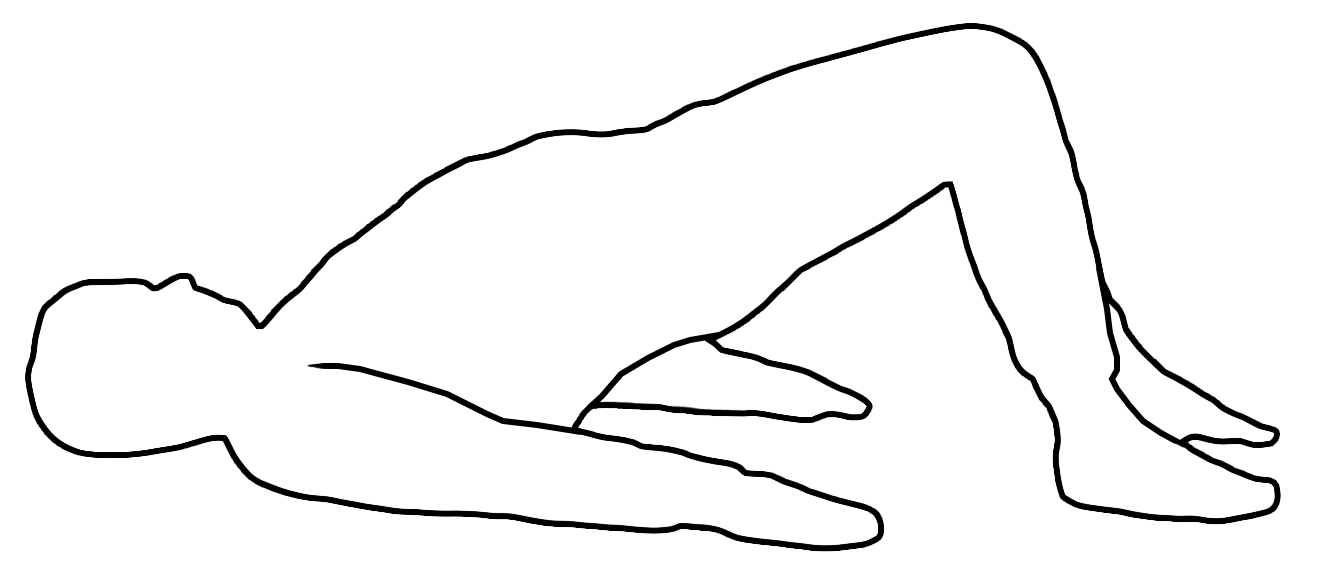
**9. Bridge.** Lie on your back with your knees bent and your legs and feet parallel and hip-distance apart. Keep your arms at your sides on the floor while you raise your pelvis as far you can comfortably and maintain a neutral lower spine. If instructed in the clinical appointment, raise your hand or leg. Inhale as you raise your pelvis and exhale as you come back to the starting position.

Repeat movement ______ repetitions ______ sets.
