# Supplementary material for: Protocol of identical exercise programs with and without specific breathing techniques for the treatment of chronic non-specific low back pain: randomized feasibility trial with two-month follow-up
Source: BMC Musculoskelet Disord. 2023 May 5;24:354. doi: 10.1186/s12891-023-06434-6 (PMC10161472; doi:10.1186/s12891-023-06434-6)
Supplement: Supplementary file 2 — Additional file 2. [file 12891_2023_6434_MOESM2_ESM.docx]

**Flexion movement control exercises**

- **Maintain a neutral lumbar spine during the exercises.**
- **Synchronize your breathing with the movements (Note: The breathing instructions were only included on the exercise sheets for group 2, otherwise the exercise sheets were identical for both groups.)**
- **Have breaks between sets and exercises according to your individual needs.**
- **Try to practice your exercises regularly/once a day.**
- **Remember to regularly update your home diary.**

**Standing exercises**


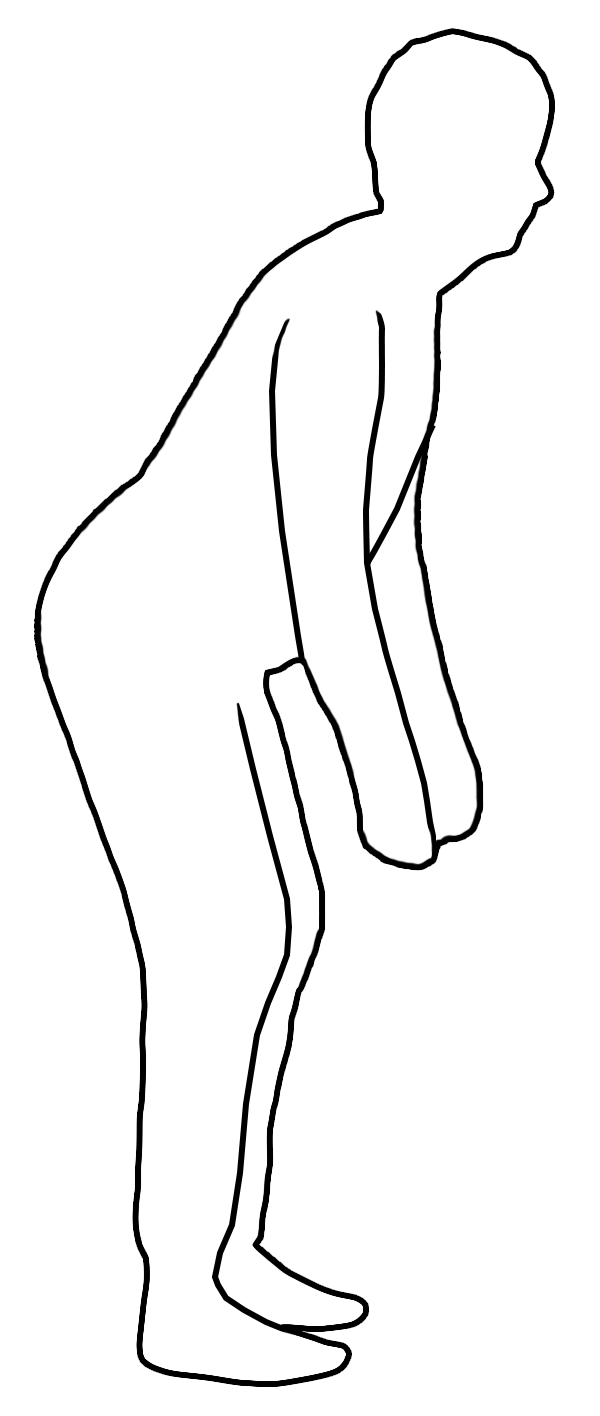


1. **Waiter’s bow.** Stand on both legs in a balanced position, with your feet separated by the same width as your hips. Bend slowly forward and down, while you maintain a neutral lower spine. You can bend your knees slightly as you bend down. Exhale as you bend forward and inhale as you return to the starting position.

Repeat movement ______ repetitions ______ sets.


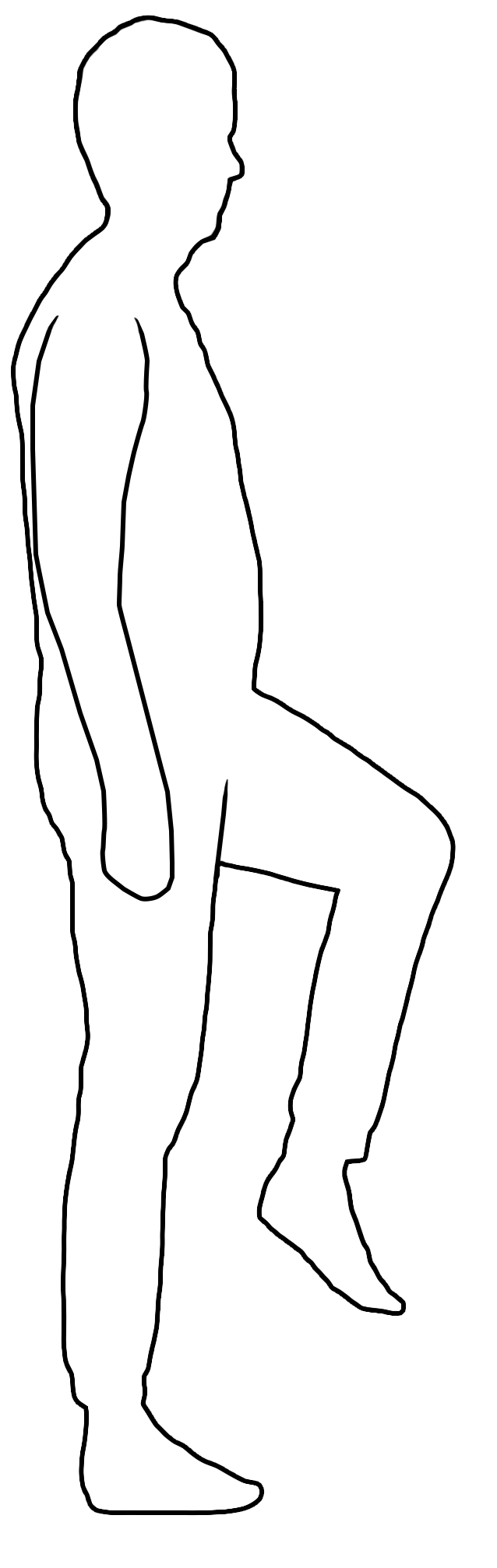


1. **One leg raise on standing.** Stand on both legs in a balanced position, with your feet separated by the same width as your hips. Then, lift one knee while maintaining a neutral spine. Inhale as you raise your knee and exhale and inhale as you return your leg to the starting position. Alternate legs between repetitions.

Repeat movement ______ repetitions with alternating legs ______ sets.


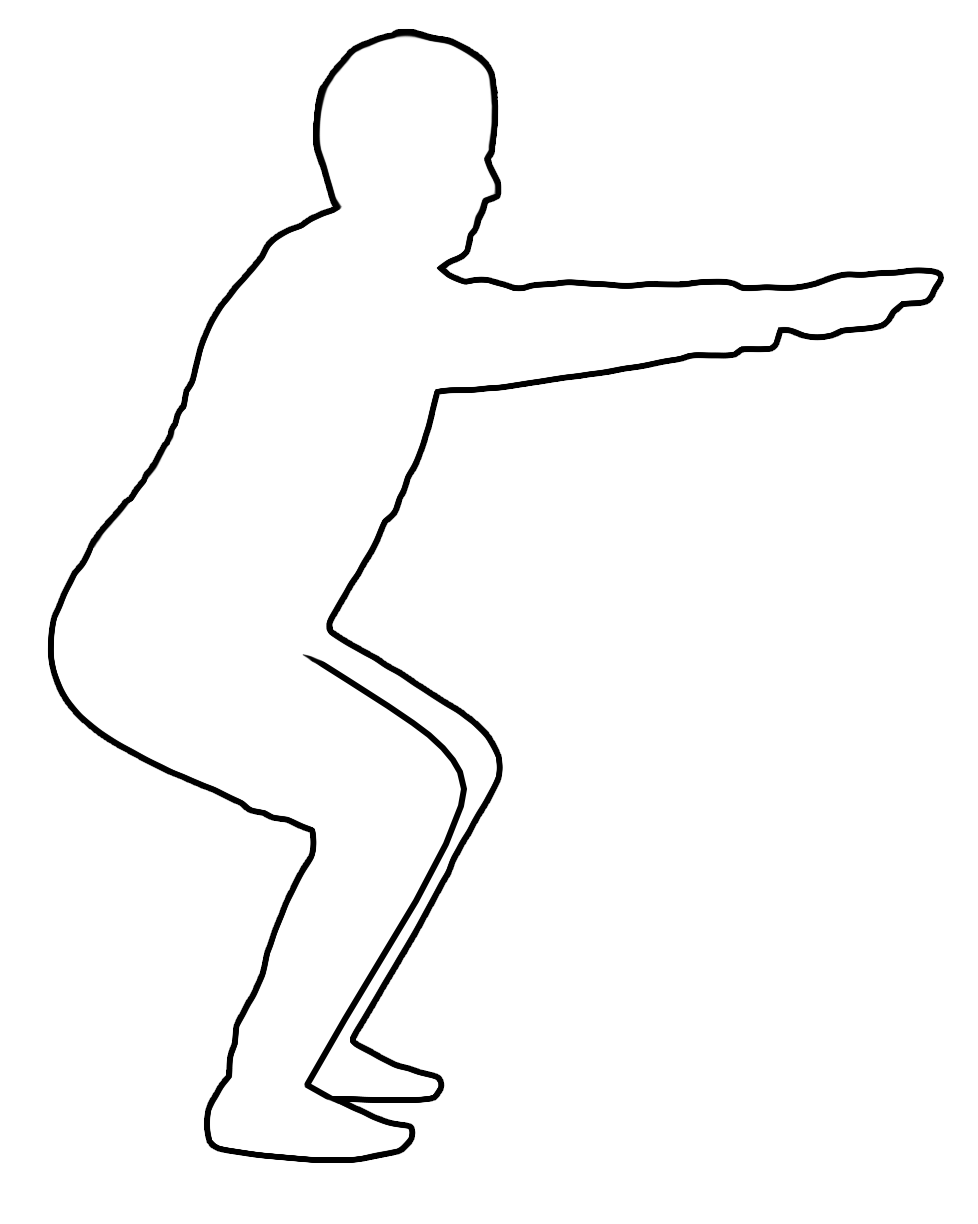


1. **Squat.** Stand on both legs in a balanced position, with your feet separated by the same width as your hips. Bend your knees and squat down as far as you can comfortably. Maintain a neutral spine as you do the squat. Exhale as you squat down and inhale as you come back to the starting position.

Repeat movement ______ repetitions ______ sets.

**Sitting exercises
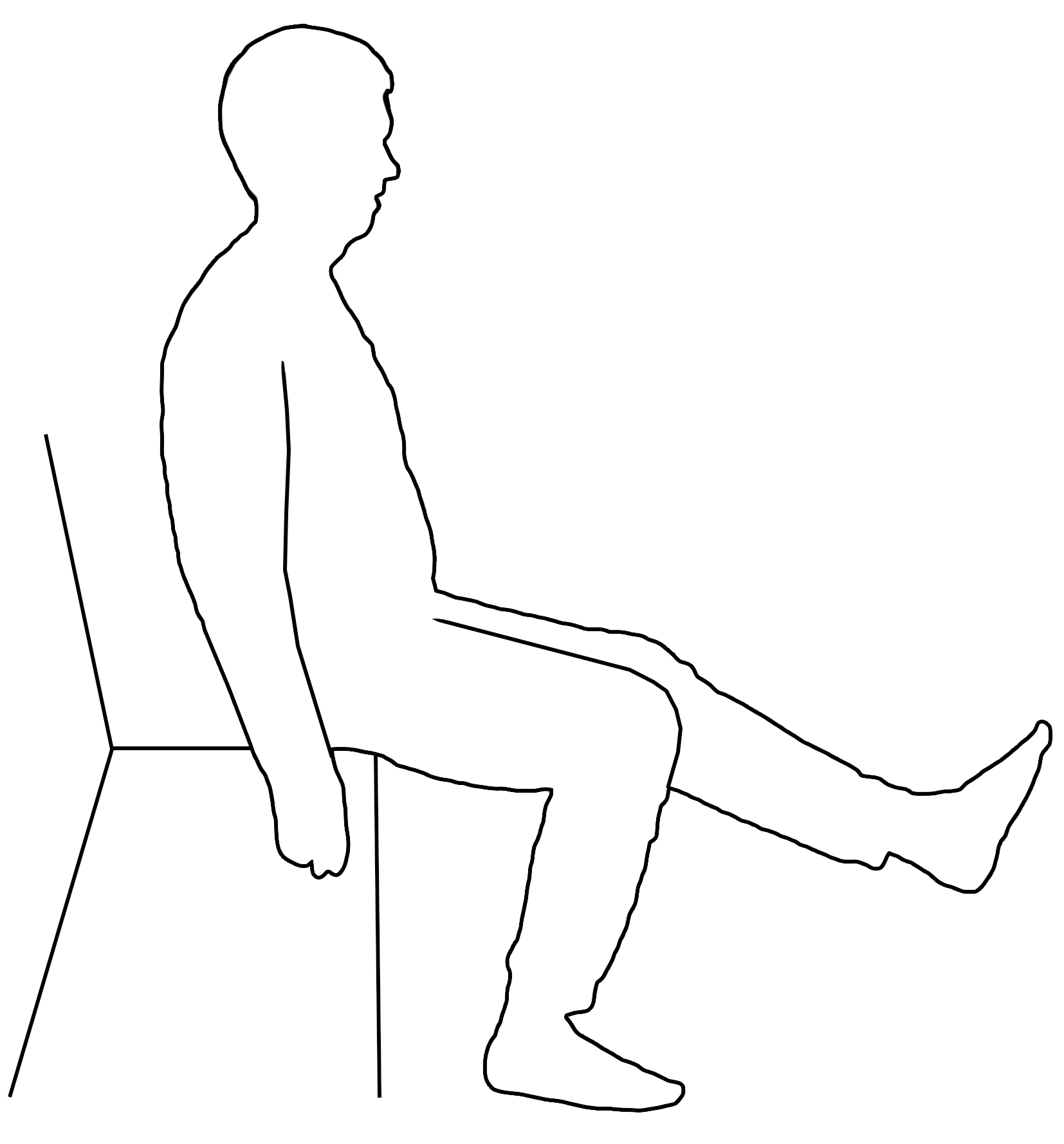
**

1. **Leg extension on sitting.** Sit in a chair. Straighten one leg while maintaining a neutral spine. Inhale as you straighten your leg and exhale as you return to the starting position.

Repeat movement ______ repetitions ______ sets alternating your legs.

**All fours exercises**

1.
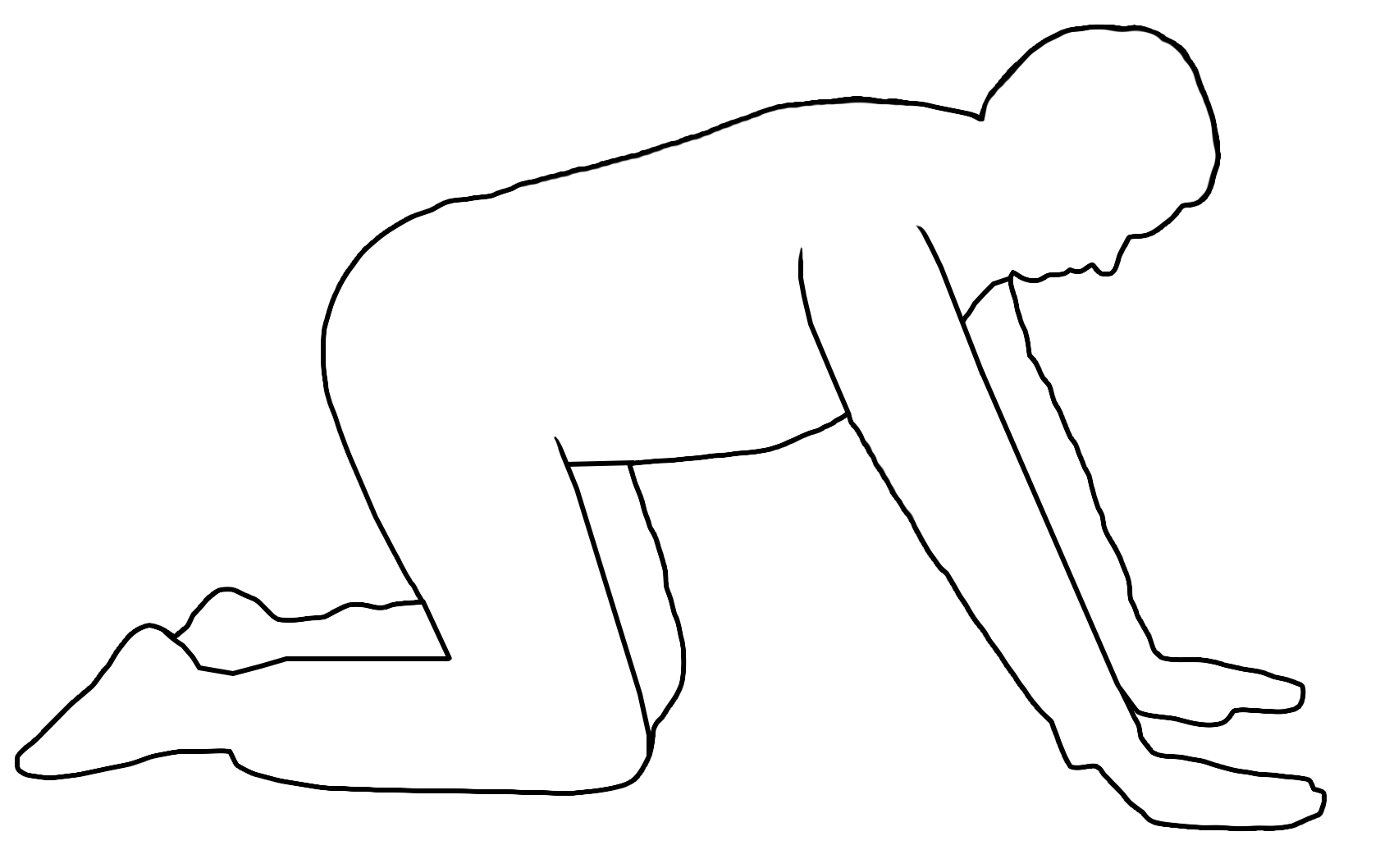
**All fours backwards.** Kneel on all fours with a neutral lower spine, with your hands and knees separated by the same width as your hips. Start to bend your knees and bring your pelvis backwards towards the floor while maintaining a neutral lower spine. Exhale as you bring your pelvis backwards and inhale as you return to the starting position.

Repeat movement ______ repetitions ______ sets.


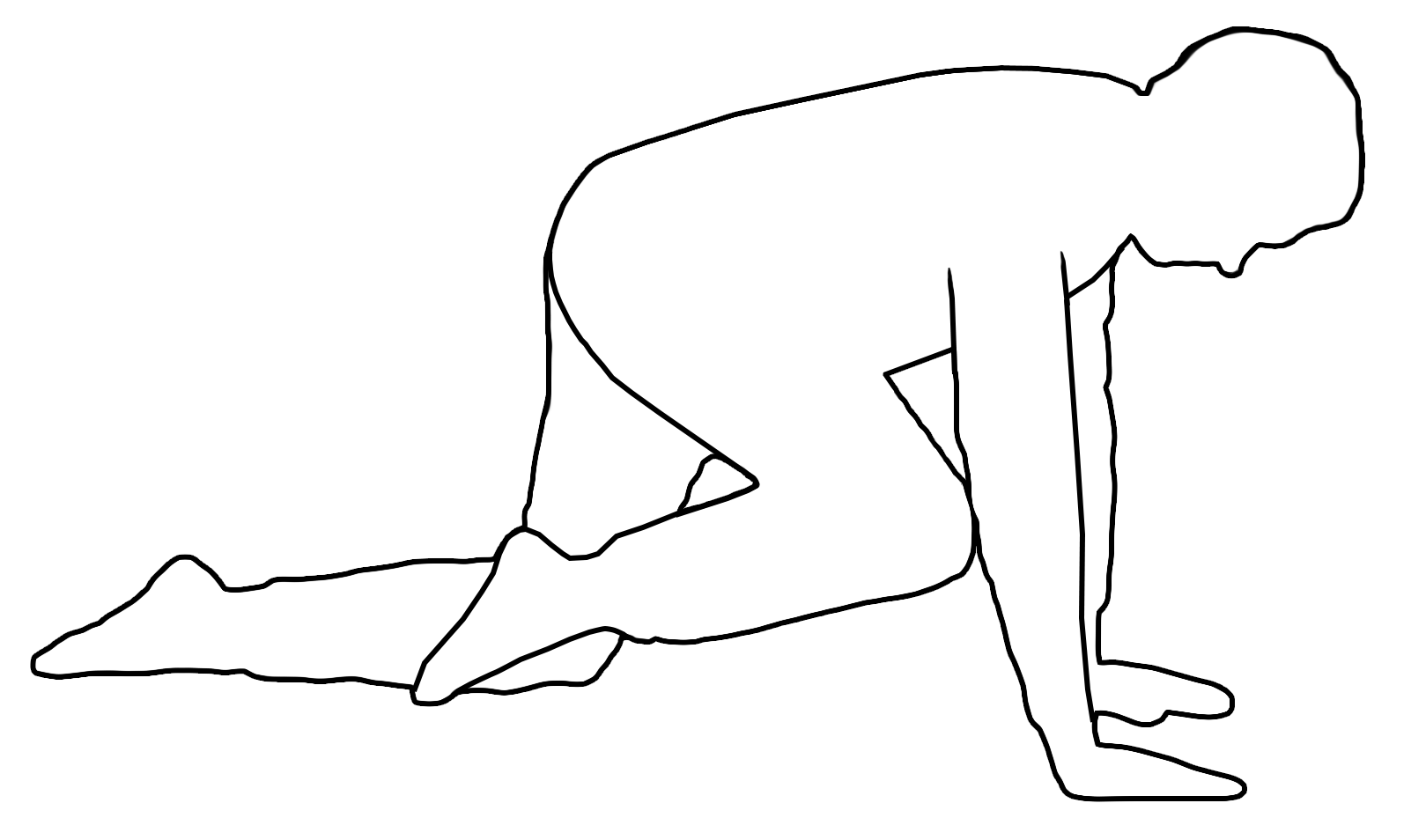


1. **All fours hip flexion.** Kneel on all fours with a neutral lower spine, with your hands and knees separated by the same width as your hips. Start to bring one knee towards your chest while maintaining a neutral lower spine. Inhale as you bring your knee towards your chest and inhale as you return to the starting position. Alternate legs between each repetition.

Repeat movement ______ repetitions ______ sets on both legs.

1.
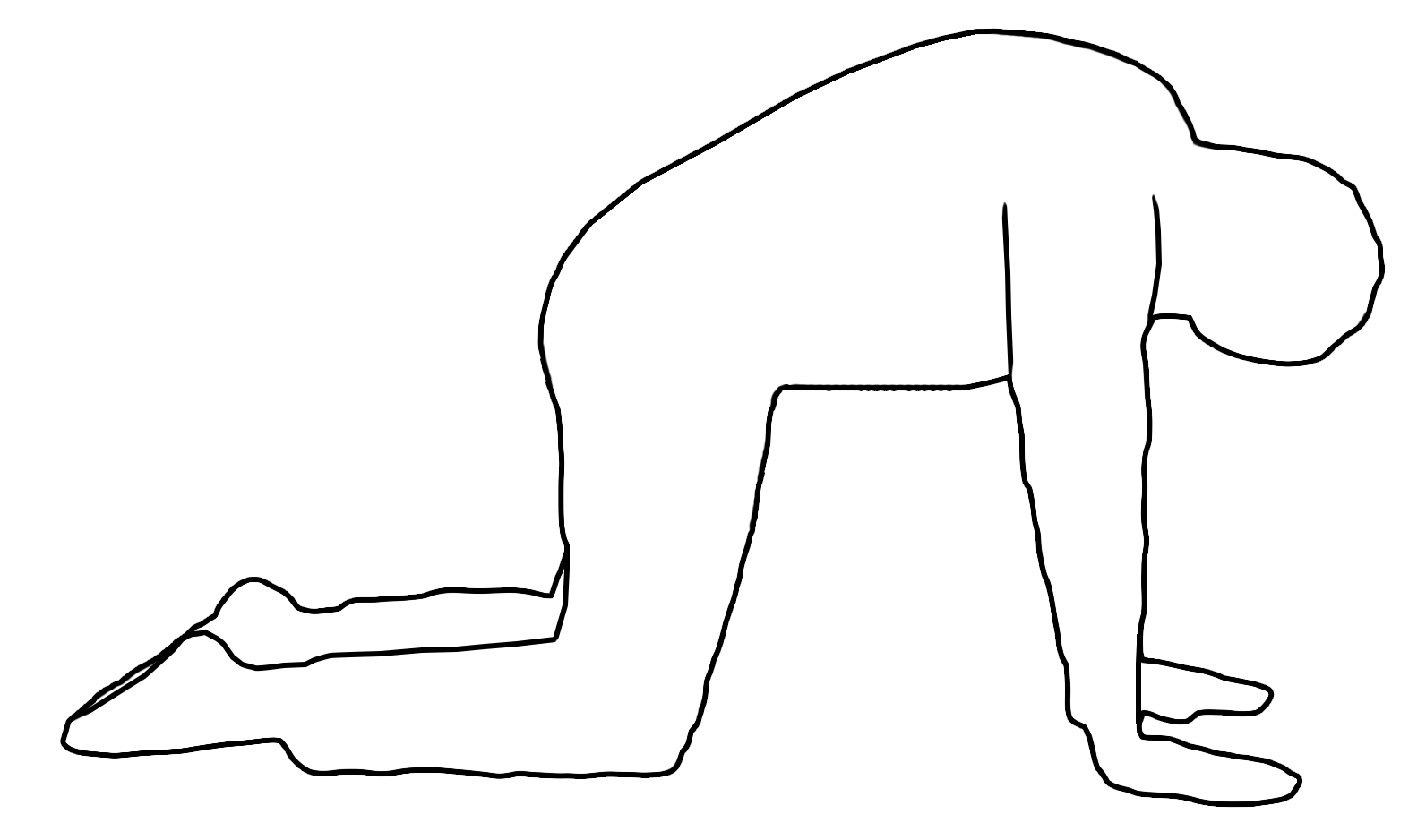
**Camel exercise.** Kneel on all fours with a neutral lower spine, with your hands and knees separated by the same width as your hips. Let your head and neck relax and gaze down, while you arch your spine upwards at same time. Exhale as you arch your spine and inhale as you come back to the starting position.

Repeat movement ______ repetitions ______ sets.

**Supine exercise**

1. **
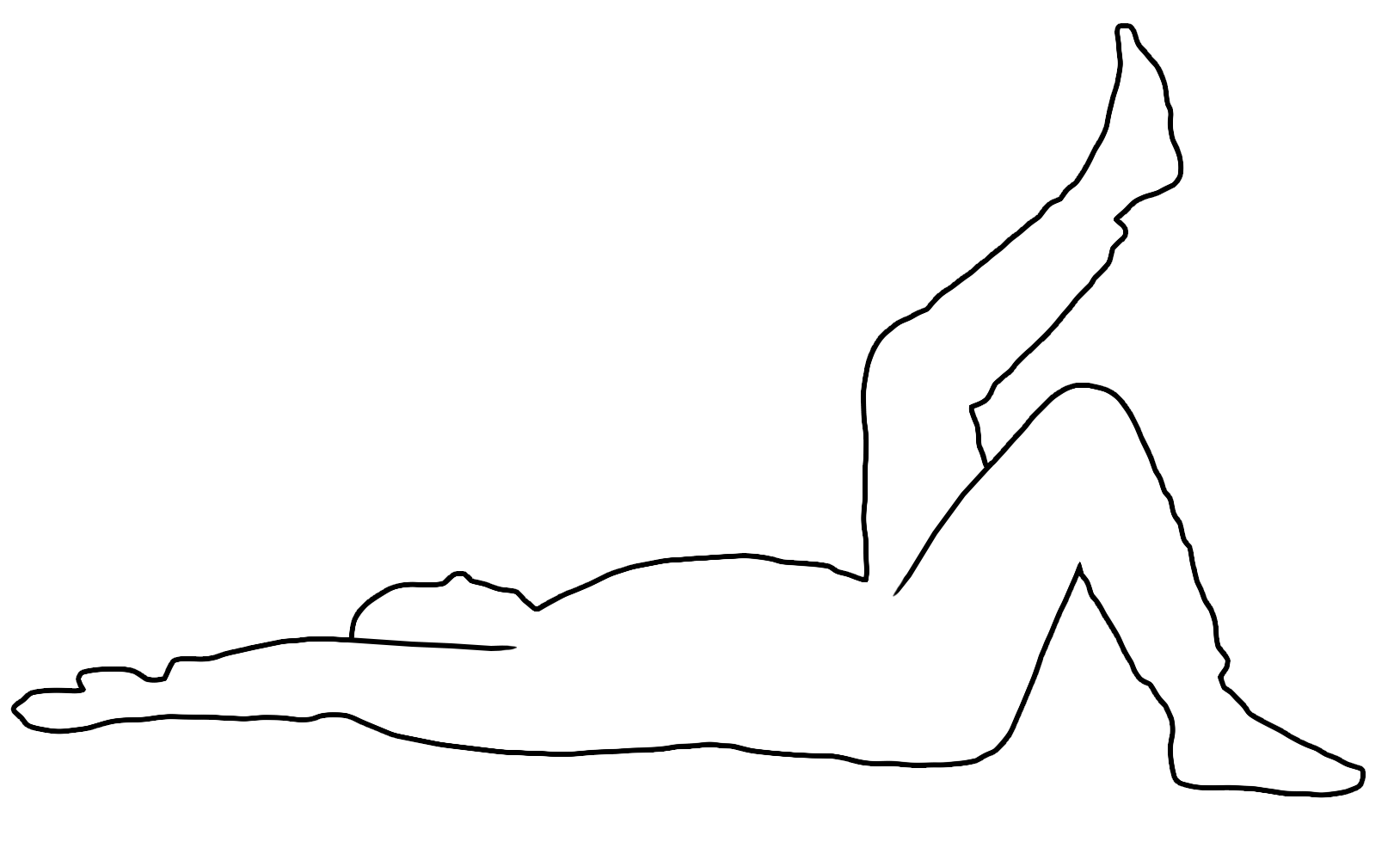
Dead bug exercise.** Lie on your back. Maintain a neutral spine as you move your leg(s) or/and arm(s) as instructed in your clinic appointment. Inhale as you move your leg(s) and/or arm(s) upwards and exhale as you come back to the starting position.

Repeat movement ______ repetitions ______ sets.

**On knees exercise**

1.
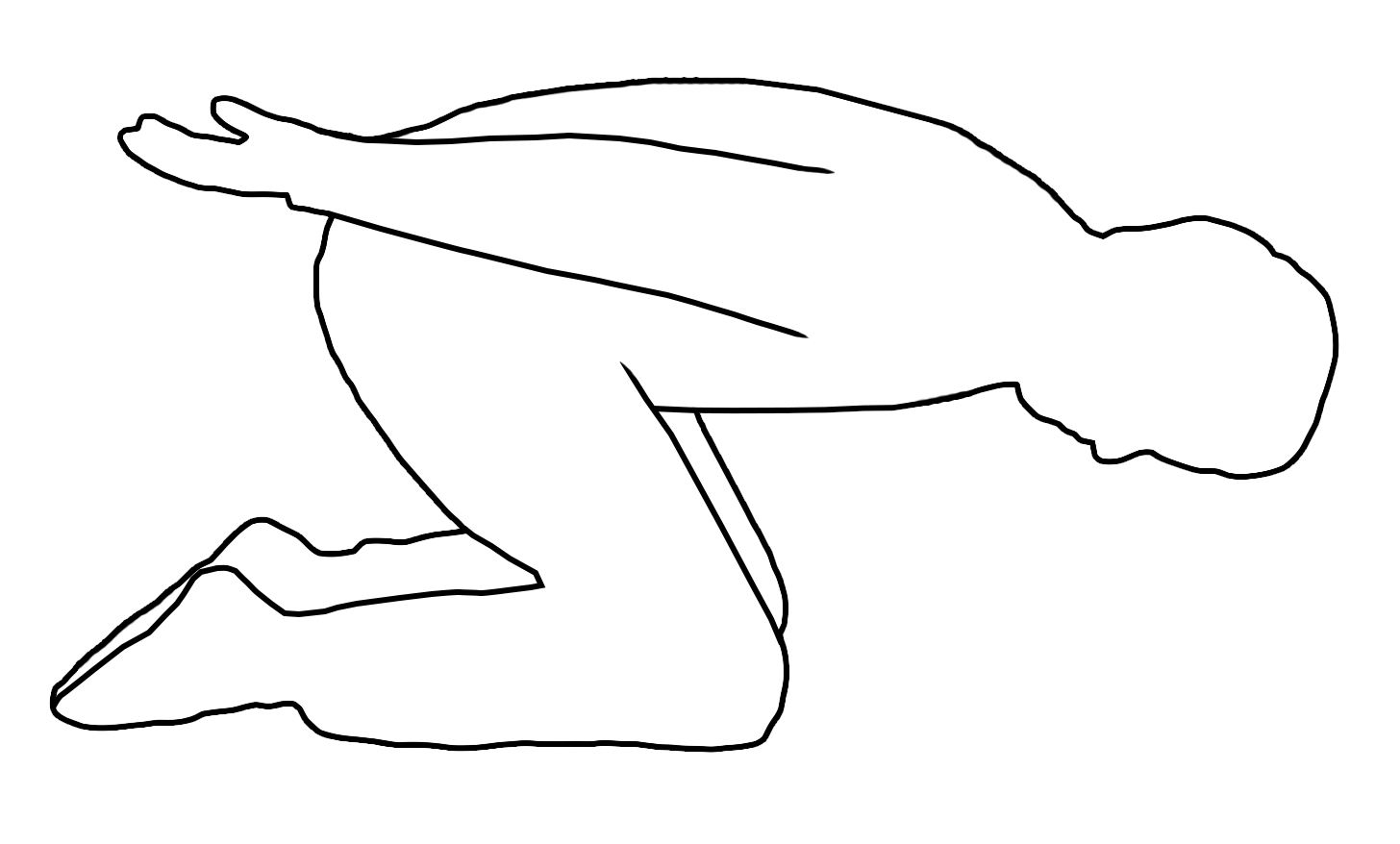
**Kneeling foward bending.** Kneel down and maintain a neutral spine. Let your knees and hips bend as you lower your forehead towards the floor; try to maintain a neutral spine at the same time. Come back up to the kneeling position. Exhale as you bend your knees and hips and inhale as you come back to the kneeling position.

Repeat movement ______ repetitions ______ sets.
