# Supplementary material for: Protocol of identical exercise programs with and without specific breathing techniques for the treatment of chronic non-specific low back pain: randomized feasibility trial with two-month follow-up
Source: BMC Musculoskelet Disord. 2023 May 5;24:354. doi: 10.1186/s12891-023-06434-6 (PMC10161472; doi:10.1186/s12891-023-06434-6)
Supplement: Supplementary file 3 — Additional file 3. [file 12891_2023_6434_MOESM3_ESM.docx]

**Rotation and sideways movement control exercises**

- **Maintain a neutral lumbar spine during the exercises.**
- **Synchronize your breathing with the movements (Note: The breathing instructions were only included on the exercise sheets for group 2, otherwise the exercise sheets were identical for both groups.)**
- **Have breaks between sets and exercises according to your individual needs.**
- **Try to practice your exercises regularly/once a day.**
- **Remember to regularly update your home diary.**

**Standing exercises**

1.
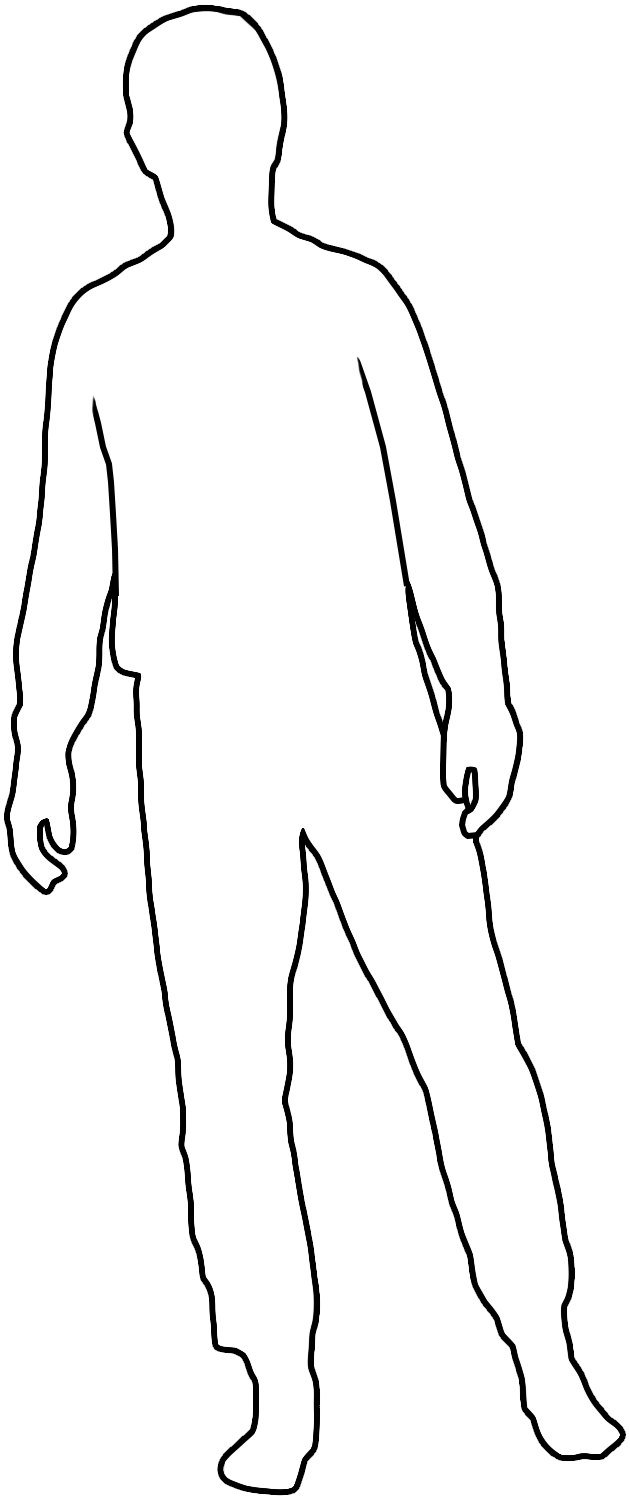
**One leg stance with weight shift.** Stand on both legs in a balanced position, with your feet separated by the same width as your hips. Start to shift your weight slowly onto one leg and gradually raise your other foot off the ground while keeping your balance. Come back to standing on both feet. Repeat this exercise with the other leg. Maintain a neutral lower spine throughout these movements. Inhale as you shift your weight and lift one leg and exhale as you return to your balance on both feet.

Repeat movement ______ repetitions with both legs ______ sets.


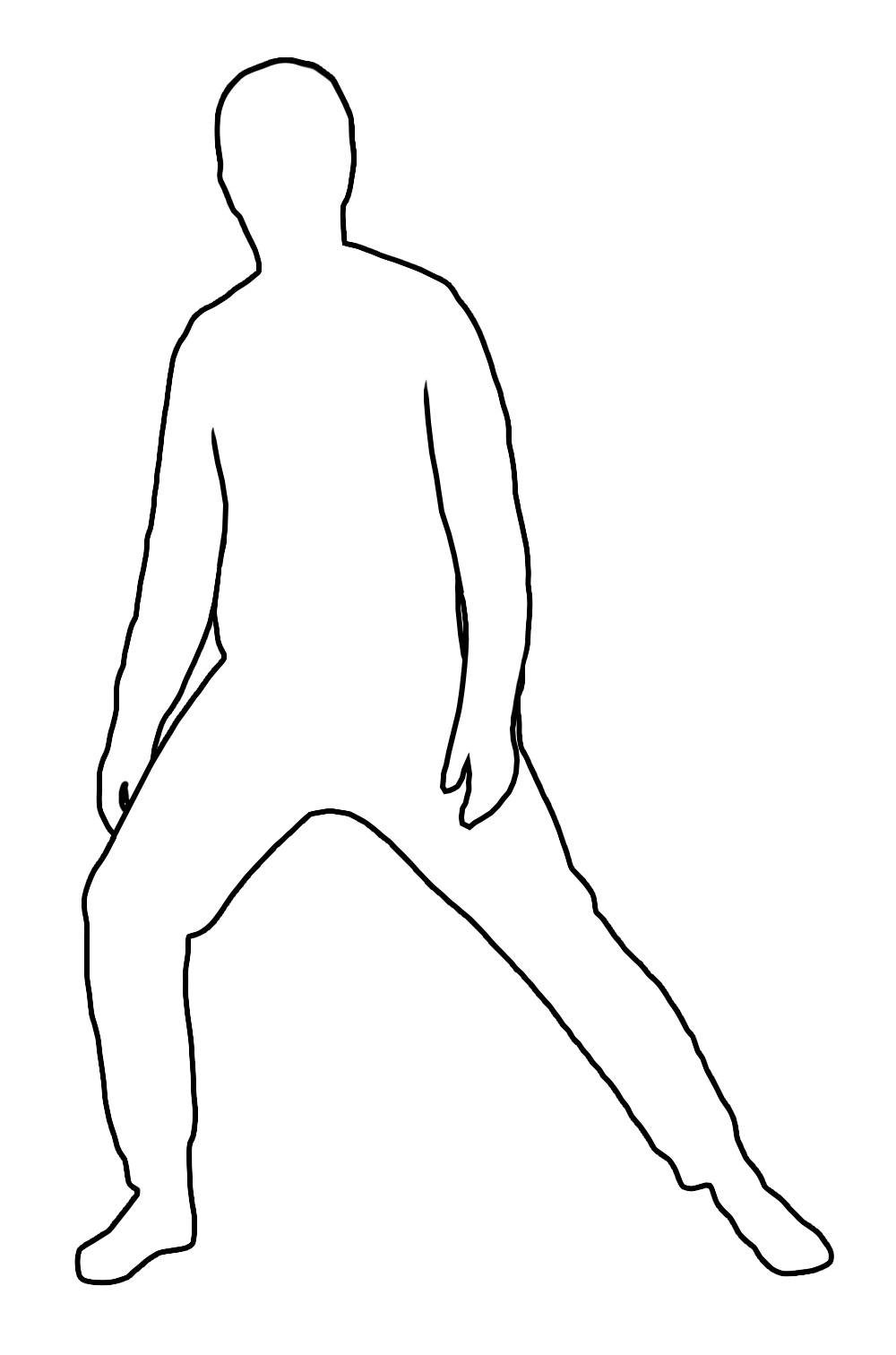


1. **Transverse lunge.** Stand on both legs in a balanced position, with your feet separated by the same width as your hips. Take a long, controlled step with one leg to the side, and let your knee bend. Step back to the starting position. Maintain a neutral spine throughout the movement. Inhale as you take the long step to the side and exhale as you bring your leg back to the middle.

Repeat movement ______ repetitions on both legs alternating leg _____ sets.


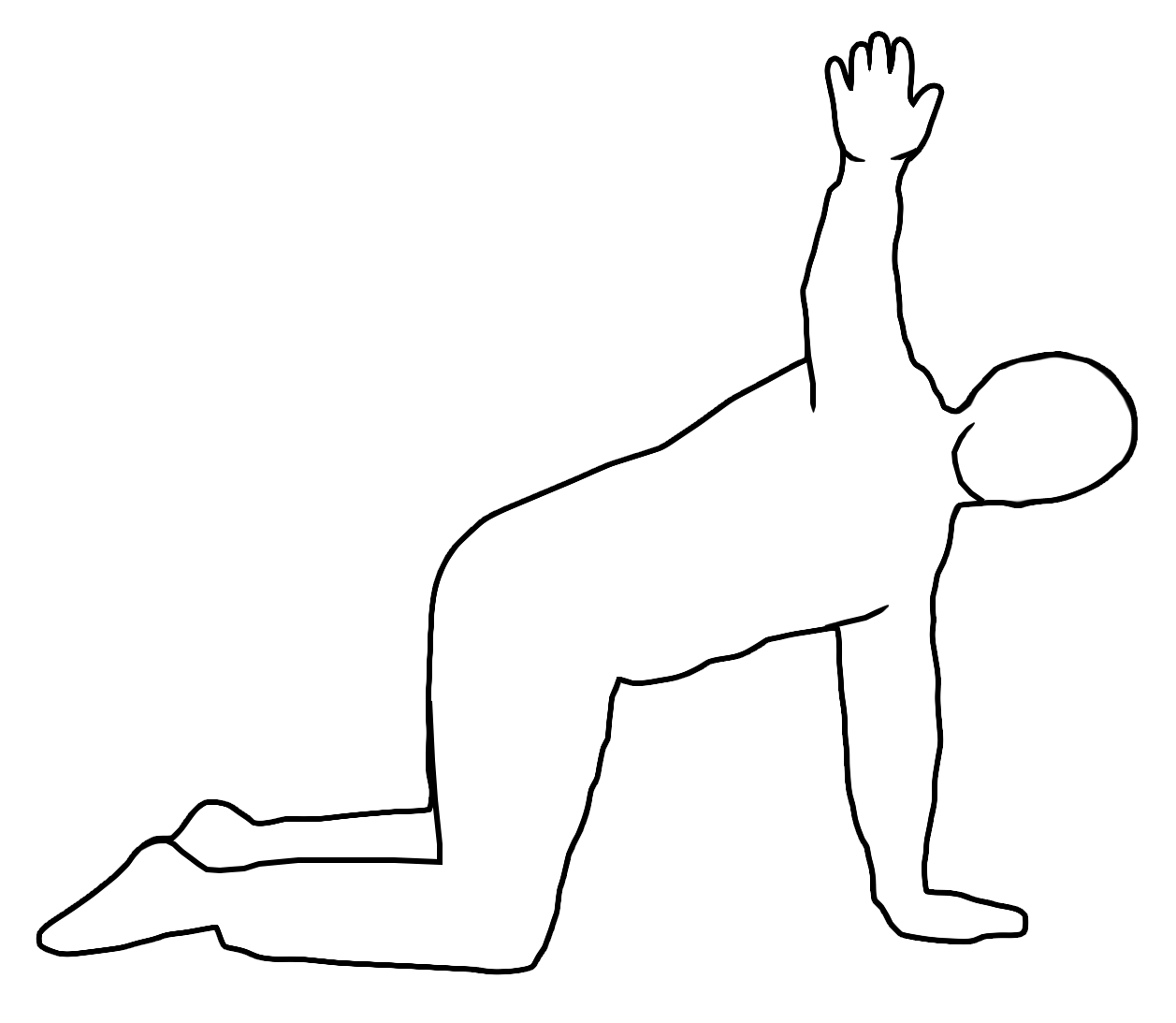
**All fours exercises**

1. **All fours rotation.** Kneel on all fours with a neutral lower spine, with your hands and knees separated by the same width as your hips. Begin to rotate your head and spine to one side and open your chest at the same time. Then move back to the starting position with a neutral lower spine. Inhale as you rotate to the side and exhale as you move back to the starting position.

Repeat movement ______ repetitions on both sides ______ sets.

1.
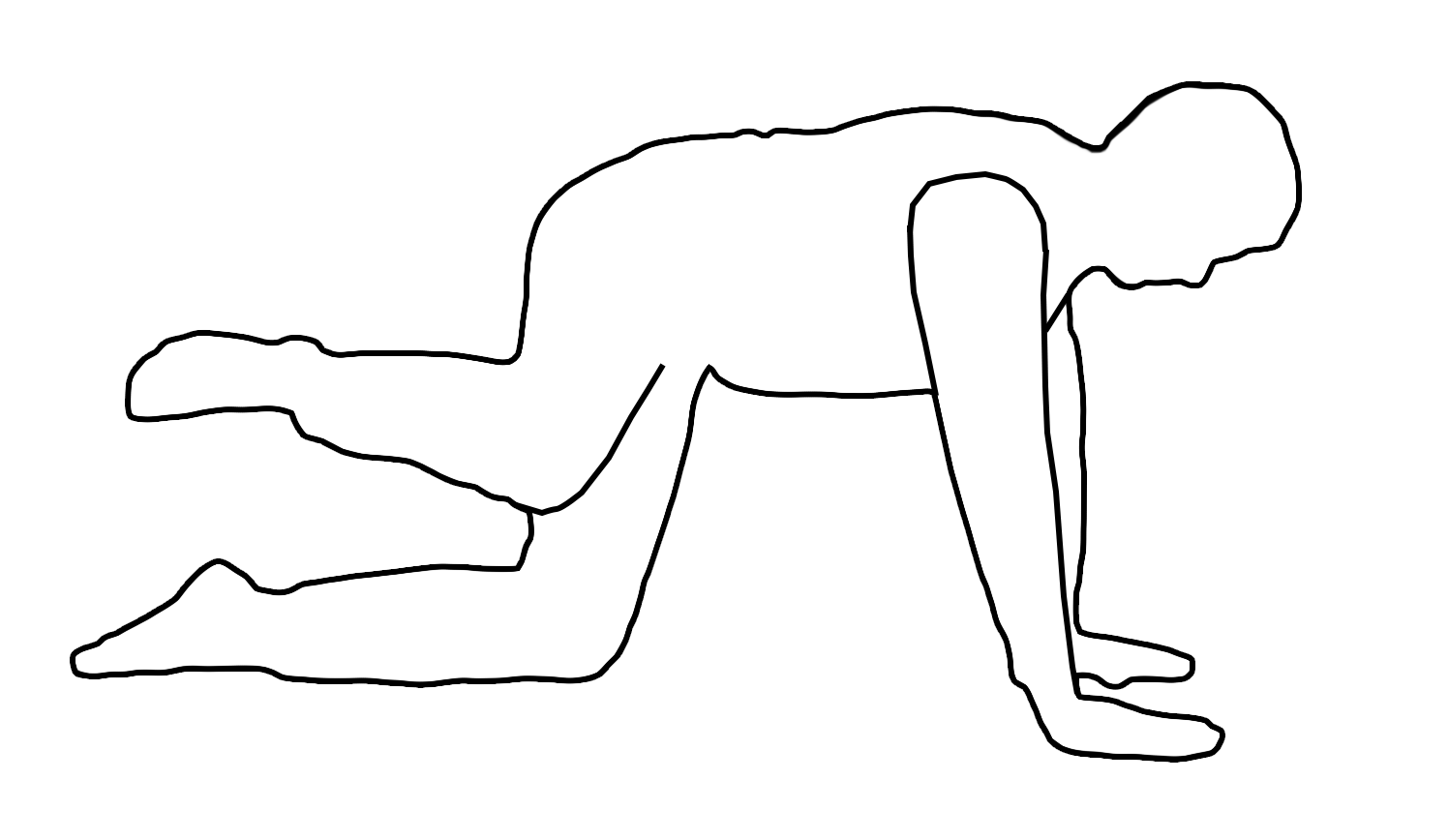
**All fours knee sideway raise.** Kneel on all fours with a neutral lower spine, with your hands and knees separated by the same width as your hips. Begin to raise one knee and leg out straight to the side as far as you can comfortably. Maintain a neutral lower spine throughout the movement. Inhale as you move your knee to the side and exhale as you bring your knee back to the starting position.

Repeat movement ______ repetitions on both sides ______ sets.

**Prone exercise**

1.
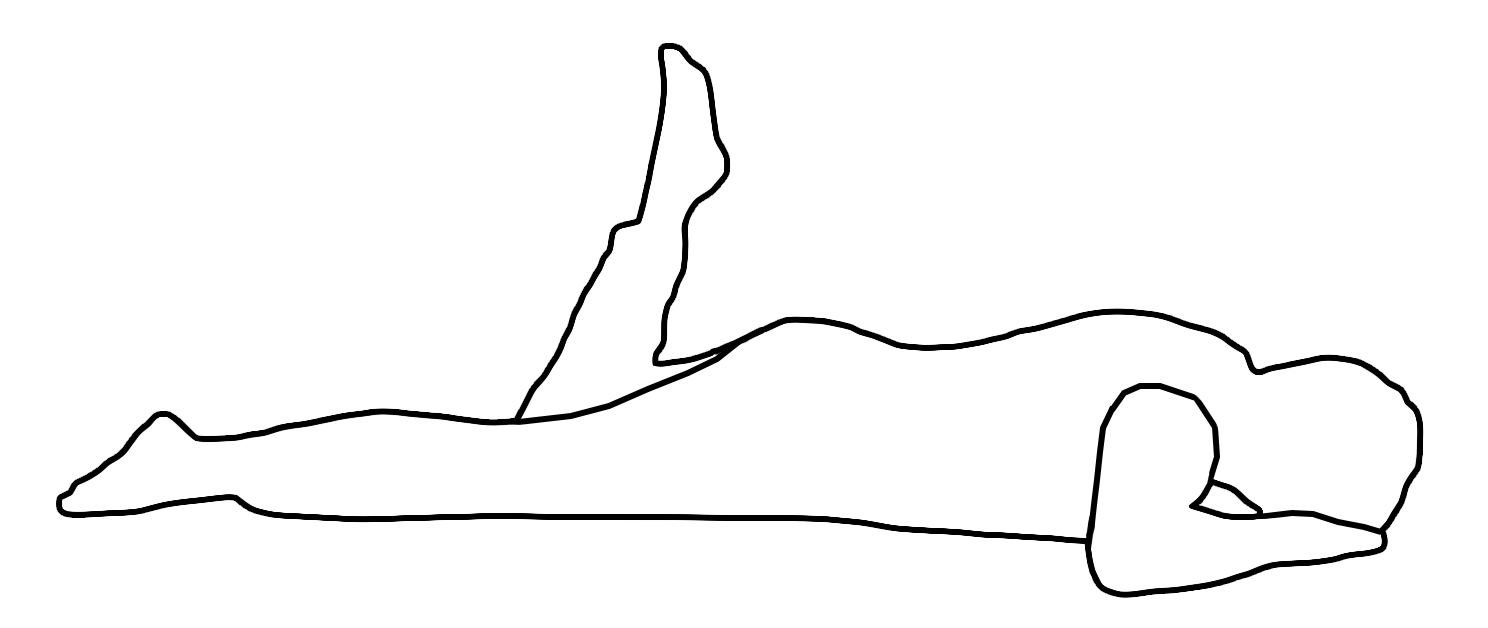
**Prone knee hip internal rotation.** Lie on your stomach and bend one knee by 90 degrees. Bring your feet to the side and maintain a neutral lower spine at the same time. Inhale as you bring your bended knee to the side and exhale as you return to the starting position.

Repeat movement ______ repetitions on both sides ______ sets.
